# Acute B lymphoblastic leukaemia-propagating cells are present at high frequency in diverse lymphoblast populations

**DOI:** 10.1002/emmm.201201703

**Published:** 2012-12-11

**Authors:** Klaus Rehe, Kerrie Wilson, Simon Bomken, Daniel Williamson, Julie Irving, Monique L den Boer, Martin Stanulla, Martin Schrappe, Andrew G Hall, Olaf Heidenreich, Josef Vormoor

**Affiliations:** 1 Newcastle Cancer Centre at the Northern Institute for Cancer Research, Newcastle UniversityNewcastle upon Tyne, UK; 2 Great North Children's Hospital, Newcastle upon Tyne Hospitals NHS Foundation TrustNewcastle upon Tyne, UK; 3 Department of Pediatric Oncology and Hematology, Erasmus MC-Sophia Children's HospitalRotterdam, The Netherlands; 4 Department of Pediatrics, University Medical Center Schleswig-HolsteinCampus Kiel, Kiel, Germany; 5 North of England Stem Cell Institute, Newcastle UniversityNewcastle upon Tyne, UK; 6 Present address: KUNO Children's Hospital, Pediatric Hematology, Oncology and Stem Cell Transplantation, University Medical Center RegensburgRegensburg, Germany

**Keywords:** acute lymphoblastic leukaemia, cancer stem cells, leukaemia maintenance, leukaemia propagating cells, leukaemia stem cells

## Abstract

Leukaemia-propagating cells are more frequent in high-risk acute B lymphoblastic leukaemia than in many malignancies that follow a hierarchical cancer stem cell model. It is unclear whether this characteristic can be more universally applied to patients from non-‘high-risk’ sub-groups and across a broad range of cellular immunophenotypes. Here, we demonstrate in a wide range of primary patient samples and patient samples previously passaged through mice that leukaemia-propagating cells are found in all populations defined by high or low expression of the lymphoid differentiation markers CD10, CD20 or CD34. The frequency of leukaemia-propagating cells and their engraftment kinetics do not differ between these populations. Transcriptomic analysis of CD34^high^ and CD34^low^ blasts establishes their difference and their similarity to comparable normal progenitors at different stages of B-cell development. However, consistent with the functional similarity of these populations, expression signatures characteristic of leukaemia propagating cells in acute myeloid leukaemia fail to distinguish between the different populations. Together, these findings suggest that there is no stem cell hierarchy in acute B lymphoblastic leukaemia.

→See accompanying article http://dx.doi.org/10.1002/emmm.201202207

## INTRODUCTION

Based on the pioneering work by Till and McCulloch (Till & McCulloch, 1961), normal haematopoiesis is known to be organized in a hierarchical fashion with a small number of multipotent stem cells maintaining all haematopoietic lineages. Similar to normal haematopoiesis, early xenotransplantation studies demonstrated that only a minority of immature blasts were able to initiate and propagate human acute myeloid leukaemia (AML) in immunodeficient mice (Bonnet & Dick, 1997; Lapidot et al, 1994). Subsequent studies have confirmed that AML is indeed maintained by rare, specialized and phenotypically defined leukaemia propagating cells (LPCs) (Goardon et al, 2011; Hope et al, 2004), even if these populations are more diverse in phenotype than originally suggested (Taussig et al, 2010).

This hierarchical cancer stem cell model has been explored in other cancer types using similar xenotransplantation models (Bomken et al, 2010). Many, including breast (Al-Hajj et al, 2003), brain (Hemmati et al, 2003; Singh et al, 2003), colon (Dieter et al, 2011; O'Brien et al, 2007; Ricci-Vitiani et al, 2007) and lung cancer (Eramo et al, 2008; Ho et al, 2007), appear to follow a similar model. However, the hierarchical stem cell model may not fit all tumours. Notably, studies with melanoma cells have shown that one in four cells, irrespective of their immunophenotype, possess the ability to propagate the tumour (Quintana et al, 2008, 2010). Therefore, the biology of tumour-propagating cells may vary between different malignancies. As shown in chronic myeloid leukaemia, secondary events during malignant progression may lead to acquisition or expansion of the cancer stem cell phenotype in malignant cells ‘downstream’ of the cell of origin (Jamieson et al, 2004). Therefore, stem cell phenotypes and frequencies may also change during tumour development and progression, consistent with the model of clonal evolution (Anderson et al, 2011; Notta et al, 2011).

In our previous work, we have challenged the hierarchical stem cell model for acute B lymphoblastic leukaemia (B-ALL) by demonstrating that phenotypically diverse blasts irrespective of the expression of the B-cell marker CD19 and the stem cell marker CD34 are able to re-establish the leukaemia in immunodeficient mice (le Viseur et al, 2008). However, key questions remained to be addressed. Here, we demonstrate that our model is more widely applicable to B-ALL by investigating different blast populations identified by the use of additional B-cell maturation markers, a wider range of ALL subtypes and, most importantly, engraftment of primary samples that have not been previously passaged through mice. For the first time, we provide quantitative data on the frequency of LPCs in different B-ALL subpopulations. We show that blasts with leukaemia-propagating capability are present at similar frequencies in all populations tested and re-grow the leukaemia with similar kinetics. In keeping with a lack of a hierarchy and the potential of all populations to propagate the leukaemia, self-renewal signatures derived from AML and normal haematopoietic stem cells fail to distinguish between the different ALL blast cell populations.

## RESULTS

### Immunophenotypic diversity and malleability of leukaemia-propagating cells

**Figure 1:**
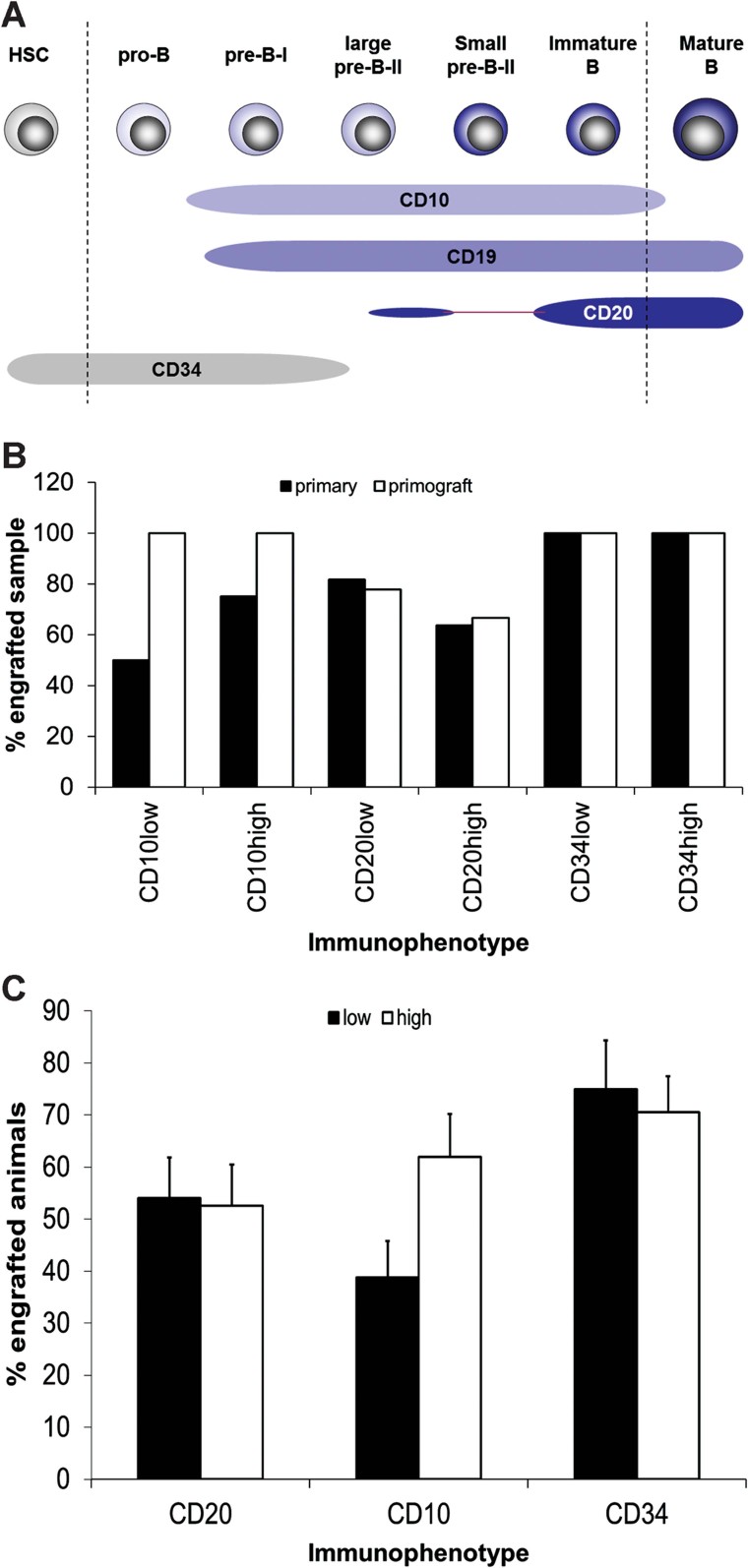
B-cell differentiation markers and engraftment potential Expression of the cell surface antigens CD10, CD19, CD20 and CD34 during normal human B-cell development (van Zelm et al, 2005).Percentage of engrafting primary patient and primograft samples for the different populations (CD10^low/high^, CD20^low/high^, CD34^low/high^). Black bars provide the data for primary and white bars for primograft samples.Weighted mean of engraftment in mice transplanted with at least 1000 blasts sorted for CD10, CD20 or CD34 expression. The analysis takes into account that different numbers of mice were transplanted with cells from different samples. For data of engraftment of mice transplanted with cells sorted for expression of CD10 and CD20, see also Supporting Information Tables S1 and S2. The CD34 data are taken from the limiting dilutions experiments (Table 2) and include only the mice transplanted with 1000 cells.

In our previous experiments, we had shown that phenotypically diverse ALL blasts, mainly characterized by expression of CD19 and CD34, are able to propagate the human leukaemia in immunodeficient mice (le Viseur et al, 2008). One question that arose from these results was whether this was a generic finding indicating the absence of a stem cell hierarchy in B-cell precursor ALL or whether other candidate B-cell markers would allow enrichment of leukaemia-propagating activity. We therefore tested CD10 and CD20, surface molecules that are expressed in a maturation-dependent fashion during normal B-cell development (Fig 1) (van Zelm et al, 2005). Importantly, the patient samples used for these experiments no longer focus on infant ALL with *MLL* rearrangements, but reflect a wider range of different ALL subtypes, including high-risk Philadelphia chromosome-positive and *BCR-ABL1-*like ALL, intermediate risk ALL with no known cytogenetic risk factors and prognostically more favourable ALL with high hyperdiploidy (Table 1). The ability of purified cells to initiate the leukaemia was interrogated by intrafemoral injection into immunodeficient NSG mice. Engrafted leukaemia mirrored the original disease with enlarged spleens and infiltration of bone marrow, liver, kidneys and the central nervous system and by morphology and immunophenotype.

**Table 1:** Patient characteristics (NCI risk factor: age and white cell count, and molecular-/cytogenetics) and experiments done with the respective samples

Patient ID	Presentation or relapse	Cytogenetics	Age at presentation	WCC (×10^9^)	Experiments
High hyperdiploidy					
L754	Presentation	High hyperdiploidy	3	142.0	2
L578	Relapse	High hyperdiploidy	6	4.1	1
L812	Presentation	High hyperdiploidy	2	81.9	7
L835	Presentation	High hyperdiploidy	4	5.8	2
4917	Presentation	High hyperdiploidy	9	13.4	3
Miscellaneous					
L736	Presentation	dic(9;20)(p13;q11)	2	71.9	2
L776	Presentation	*IGH2-CRLF2*	4	50.8	2,7
L784	Presentation	dic(9;20)(p13;q11)	18	52.2	1,6,8
L787	Presentation	Normal^a^,^b^	19	11.6	7
L803	Presentation	Other	18	99.7	1
L825	Presentation	Normal^a^,^b^	14	122	3
L831	Presentation	Fail^a^,^b^	16	6.1	1,2
L833	Presentation	Other^a^	14	162	2
L858	Presentation	Normal^a^,^b^	14	27.0	3
*t(4;11)(q21;q23)* or other MLL rearrangements					
L826	Presentation	t(4;11)	<1	>50	3,8
L876	Presentation	t(4;11)	<1	258	3
L727	Presentation	t(11;19)(q23;p13.3)/*MLL-ENL*	1	94	2
*t(9;22)(q34;q11)* and BCR-ABL1-like					
L4945	Presentation	t(9;22)	7	82	2
L4951	Presentation	t(9;22)	15	184	1,2,3,5,6,8
L4967	Presentation	t(9;22)	13	230	2,3
L49101	Presentation	t(9;22)	3	55.9	1,2,3,8
L49120	Presentation	t(9;22)	16	66.2	1,3
2003	Presentation	t(9;22)	15	179	3
2510	Presentation	t(9;22)	16	44.2	1,2,5,8
4540a	Presentation	t(9;22)	2	71.0	6
8849	Presentation	t(9;22)	20	360	1
EMCR1	Presentation	*BCR-ABL1-*like	4	264	1,2,3,4,5,6,8
EMCR2	Presentation	*BCR-ABL1-*like	5	97.5	1,2,3

Symbols used for additional molecular diagnostics are:

a no *ETV6-RUNX1*, no *BCR-ABL1* and no *MLL* rearrangement;

b no *IZKF* deletion.

The codes for the experiments are:

1:transplantation of blasts sorted for CD10; 2: transplantation of blasts sorted for CD20; 3: transplantation at limiting dilution with unsorted cells; 4: CD10 limiting dilution; 5: CD20 limiting dilution; 6: CD34 limiting dilution; 7: expression arrays with cells sorted for CD34; 8: *TERT* expression in blasts sorted for CD34.

To summarize our experiments, blasts from eight primary patient samples and five samples that have previously been passaged through the mice (primografts; of which four were unrelated to the primary samples) were sorted for low and high expression of CD10 (Supporting Information Table S1). These cells were injected intrafemorally into 134 NSG mice. Purified blasts from six primary and all primograft samples engrafted. Overall, both CD10^high^ and CD10^low^ populations from four of the six primary and from each of the five primograft samples were able to transfer the leukaemia onto the mice.

Similarly, blasts from 11 primary patient samples and 9 primografts (5 of these primografts derived from one of the 11 primary samples and 4 from unrelated samples) were sorted for low and high levels of CD20 expression and transplanted into 400 immunodeficient NSG mice (Supporting Information Table S2). Purified blasts from 10 primary and 7 primograft samples engrafted. Overall, both CD20^high^ and CD20^low^ populations from 8 of the 10 different primary and 6 of the 7 primograft samples were able to transfer the leukaemia onto the mice. Extending our previous data (le Viseur et al, 2008), these results confirm that in the majority of samples, representing a wide range of B-ALL subtypes, all the different immunophenotypes harbour leukaemia-propagating potential (Fig 1B–C). Most importantly, we show that this phenotypic diversity of leukaemia-propagating cells in B-ALL not only applies to cells that have been passaged through mice, but also to primary patient samples.

**Figure 2:**
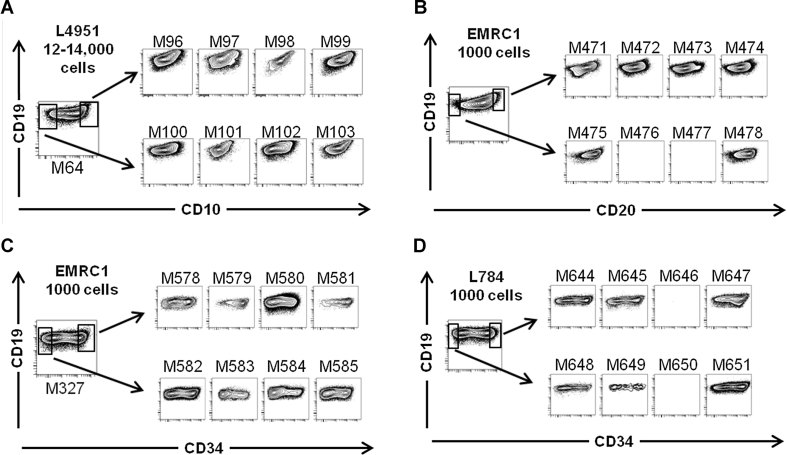
Phenotype of the re-established leukaemia after transplantation of purified B-ALL blast populations Engraftment of primografted CD10^low^ and CD10^high^ cells (patient L4951).Engraftment of primografted CD20^low^ and CD20^high^ cells (patient EMCR1).Engraftment of primografted CD34^low^ and CD34^high^ cells (patient EMCR1).Engraftment of primary CD34^low^ and CD34^high^ cells (patient L784).

We have previously suggested a model of malleability whereby different B-ALL populations sorted for a specific surface marker could re-establish their phenotypic counterparts *in vivo*. Here, we confirm this malleability for the B-cell differentiation markers CD10, CD20 and CD34 in mice transplanted with purified cells at low cell doses. Fig 2A–C shows the flow analysis of leukaemias initiated by blasts sorted for high and low expression of CD10, CD20 or CD34. All populations were able to reconstitute their corresponding population *in vivo*. The same picture was observed in mice transplanted with cells purified from primary samples: CD20^low^ and CD20^high^ as well as CD34^low^ and CD34^high^ cells (Fig 2D) engrafted and were able to reproduce both populations.

### Correlation of CD34 status with B-cell maturation-associated gene expression signatures

One of the challenges in identifying different stages of B-cell maturation in ALL is that expression of candidate B-cell differentiation markers is often homogenous and does not follow the coordinated pattern of expression seen in normal B-cell development. The aberrant expression of surface markers allows characterization of leukaemia-associated immunophenotypes that are used clinically to monitor disease (Irving et al, 2009). This may simply reflect the fact that the malignant transformation has altered the normal lymphoid differentiation programme and leukaemic stem cells may have a phenotype different to that of their normal counterparts. However, the inability to identify populations by marker combinations that correspond to the well-defined stages of pro-B to pre-B maturation raises the question about the relevance of the expression levels of individual surface markers in a malignant context.

We had previously shown that down-regulation of CD34 expression correlates with increased expression of candidate B-cell differentiation genes (*IRF4, MS4A1*/CD20, IgH constant locus, IgL *κ* & *λ* loci; le Viseur et al, 2008). To further investigate whether, and to what extent, these populations mirror different stages of normal B-cell differentiation, we compared gene expression pattern of primary B-ALL cells sorted for high and low expression of CD34 with those from CD34-positive human haematopoietic stem and progenitor cells and cells of the B-lymphoid lineage. We generated a 33-gene signature comprising of the most consistently differentially expressed genes between matched CD34^high^ and CD34^low^ leukaemic populations with more than threefold differences in expression and >100-fold difference in absolute intensity in each patient (Supporting Information Table S3). Many genes in this signature including *BCL6, BACH2, MS4A/CD20* and several immunoglobulin genes are known to be related to B-cell differentiation (Supporting Information Fig S1A). The signature is notably devoid of traditional leukemic self-renewal genes, *e.g.* the Hox gene cluster. Principal component analysis (PCA) using this 33-gene signature separated not only CD34^high^ blasts from three different B-ALL patients from the corresponding CD34^low^ blasts, but also normal CD34^high^ haematopoietic stem and progenitor cells from more differentiated B-cell stages. Consequently, CD34 status confers some real transcriptional differences between subpopulations and these directly mirror and reflect lymphoid maturation (Supporting Information Fig S1B). Gene Set Enrichment Analysis (GSEA) ranking genes according to their relative expression between CD34 subpopulations identified many significantly enriched gene sets reflecting the transition from immature to mature B cells (Supporting Information Table S4). In a reverse experiment, we designed a customized 204-gene set containing genes differentially expressed between normal pro-B and mature B cells (see the Materials and Methods section) and applied it in GSEA and found that this gene set was significantly enriched with respect to differential expression between CD34^high^ and CD34^low^ B-ALL patient cells (Supporting Information Fig S1C and Supporting Information Table S5), providing further evidence that the CD34^high^ and CD34^low^ subpopulations exhibit a real difference in expression, which recapitulates to a measurable extent B-cell maturation and support our model of malleability.

### Regulatory networks and candidate stem cell genes in B-ALL

Our xenotransplantation data suggested that blasts sorted for different surface markers should express key components of cellular programmes that are essential for a sustained, if not unlimited, proliferative potential.

**Figure 3:**
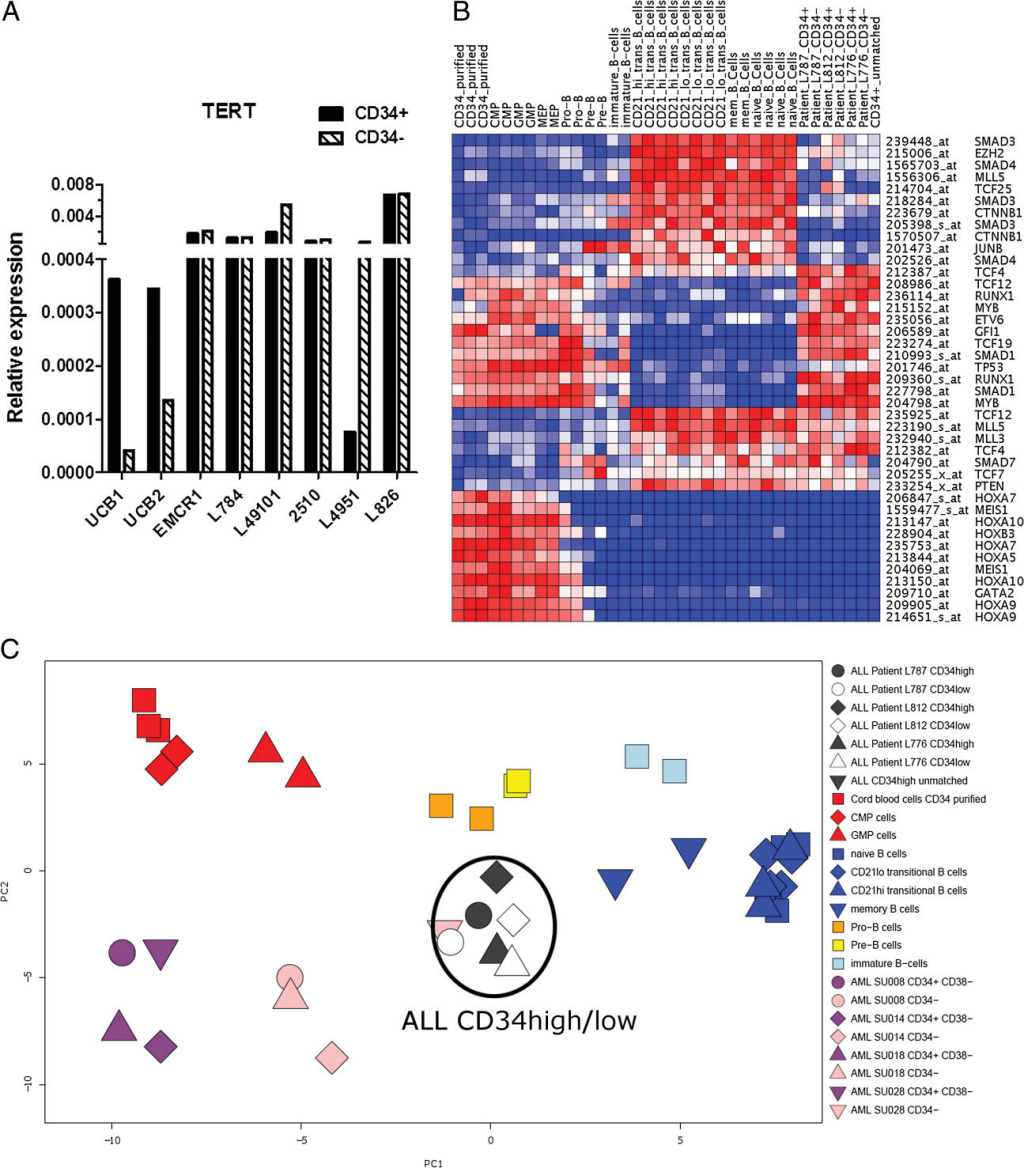
Self-renewal gene expression in CD34^high^ and CD34^low^ B-ALL blasts Expression of TERT in CD34^high^ and CD34^low^ umbilical cord blood cells and leukaemic blasts. Umbilical cord blood shows higher expression of *TERT* in immature CD34^high^ cells whilst no difference is seen between immature CD34^high^ and CD34^low^ blasts. *TERT* expression in blasts is comparable with, or higher than, that in CD34^high^ umbilical cord blood progenitor cells. Expression values are 2^-ΔCt^ using *GAPDH* as the reference gene. For purity of cells sorted for CD34 expression, see Supporting Information Fig S6.The PCA using a haematopoietic “self-renewal” signature containing 59 genes (213 probes) (Kim et al, 2009) also failed to separate CD34^high^ and CD34^low^ B-ALL populations (Supporting Information Fig S4). Here, we show the heatmap of the expression of 41 selected probes from this “self-renewal” signature.PCA plot generated using an AML “stem cell” signature (Eppert et al, 2011) that separates Lin-CD34^high^CD38^low^ blasts (dark purple symbols) from more mature CD34^low^ (pink symbols) blasts in AML (Gentles et al, 2010) but fails to detect any significant differences in “self-renewal” gene expression amongst the leukaemic subpopulations (black & white symbols) in B-ALL.

The candidate gene we chose to test this hypothesis was *TERT,* encoding the reverse transcriptase protein subunit of telomerase. Telomerase restores telomeres and prevents replicative senescence. TERT protein supports the maintenance and expansion of normal and cancer stem cells both by telomerase-dependent and -independent mechanisms (Stewart et al, 2002). Moreover, our previous experiments showed that its expression is induced and maintained by key leukaemic fusion oncogenes, supporting its significant role in leukaemic propagation (Gessner et al, 2010). Unfortunately, the low expression levels of *TERT* may impede detection by gene expression arrays. We, therefore, analysed expression of *TERT* in CD34^high^ and phenotypically more mature CD34^low^ blasts using quantitative RT-PCR. In the absence of telomerase-independent alternative lengthening of telomeres, only blasts with *TERT* expression should possess the ability for long-term leukaemia propagation. As predicted, *TERT* mRNA levels in normal human umbilical cord blood were approximately five times higher in immature CD34^high^ as compared with mature CD34^low^ cells. In contrast, there were no differences in *TERT* expression between CD34^high^ and CD34^low^ leukaemic blasts (Fig 3A).

In hierarchically organized murine AMLs, it has been possible to define a stem cell-specific core transcriptional programme expressed by the leukaemic stem cells, which is responsible for the maintenance of the leukaemic stem cell pool (Somervaille et al, 2009). In human AML, similar stem cell expression programmes have been derived from phenotypically defined candidate leukaemic stem cell populations (Gentles et al, 2010) and from cell populations with the ability to repopulate xenotransplanted mice (Eppert et al, 2011). Both signatures have been shown to correlate with clinical outcome.

To test our hypothesis that the regulatory network that describes leukaemic propagation in B-ALL is different to the stem cell programme in a hierarchical system, we performed a PCA as before but this time using Eppert's 42-gene (48-probe) signature derived from candidate human AML stem cell populations capable of repopulating immunodeficient mice (Eppert et al, 2011). Component 1 represents the transition from undifferentiated self-renewing haematopoietic progenitors to lineage-restricted B lymphoid cells (Fig 3C). Component 2 represents the difference between leukaemic and normal cells. As expected, this AML repopulating cell gene signature correctly encapsulates the difference between immature Lin-CD34^high^CD38^low^ and phenotypically more mature CD34^low^ AML blasts (Gentles et al, 2010) (purple and pink symbols), but fails to distinguish CD34^high^ from CD34^low^ blasts in B-ALL (black and white symbols), indicating the fundamental similarity of the two populations in relation to expression of candidate stem cell genes. The inability of stem cell signatures derived from AML and normal haematopoiesis to distinguish between phenotypically more immature and more mature populations in ALL was independently confirmed using a second AML leukaemic stem cell signature (Gentles et al, 2010), a normal human haematopoietic stem cell signature derived from NSG mouse engrafting populations (Eppert et al, 2011) and a haematopoietic ‘self-renewal’ signature containing 59 genes (213 probes) derived from the transcriptome of CD34^high^ haematopoietic stem/progenitor cells (Kim et al, 2009) (Supporting Information Figs S2–S4). Unlike the analysis reflecting B-cell differentiation (Supporting Information Fig S1), the PCAs using normal and AML ‘stem cell’ signatures showed no distinction between CD34^high^ and CD34^low^ B-ALL blast populations.

The uniform expression of candidate stem cell genes in CD34^high^ and CD34^low^ B-ALL blast populations is also illustrated in the heatmaps displaying selected probes from the self-renewal signature described by Kim and co-workers (Kim et al, 2009; Fig 3B). As seen previously for *TERT*, several candidate genes that have been implicated in the self-renewal of leukaemic stem cells, such as *MYB* (Broske et al, 2009; Zuber et al, 2011), are expressed both in immature and more mature B-ALL blasts (Fig 3B). These analyses are consistent with the ability of the different lymphoid populations to propagate the leukaemia in immunodeficient mice. It should be noted that there are also significant differences in expression of certain genes between B-ALL blasts and normal hematopoietic and AML stem cell populations (Fig 3B). As might be expected, given the absence of *MLL* rearrangements in the ALL cohort, the most prominent difference was the absence of expression of the *HOX* gene cluster both in CD34^high^ and CD34^low^ B-ALL populations. These differences in relation to the expression of self-renewal genes are also reflected in the multicomponent analyses in which neither the ALL blasts nor mature B lineage cells with the potential for clonal expansion cluster together with candidate AML stem cells (Fig 3C) or normal haematopoietic progenitor cells (Fig 3C; Supporting Information Figs S2–S4).

In summary, this analysis shows that the two different CD34^low^ and CD34^high^ leukaemia-propagating subpopulations of B-ALL are indistinguishable with respect to several published self-renewal signatures and that the programme underlying clonal expansion in B-ALL shows only a limited overlap with the stem cell programme of normal haematopoietic and AML stem cells.

### High frequency of leukaemia-propagating cells across phenotypically diverse blasts

If there is no hierarchy in B-ALL, as suggested by our functional analysis, and given the right environmental/niche signals, in principal every blast should have the potential to re-grow and propagate the leukaemia. Even when taking the pro-apoptotic effects of *ex vivo* handling of B-ALL blasts and the potentially hostile xeno-environment in the mice into account, the frequency of blasts with leukaemia-propagating potential should be high and equal in the different populations.

Using improved, more immunodeficient mouse strains, namely NSG mice, leading to increased sensitivity of the xenograft model, there is now a more realistic opportunity to estimate the frequency of human LPCs. Initial data on a limited number of B-ALL samples (*n* = 5) had suggested that human LPC may be more frequent than previously expected (Morisot et al, 2010). To further investigate LPC frequencies in B-ALL we transplanted blasts from 11 primary diagnostic B-ALL samples that have been previously shown to engraft NSG mice and three additional primografted B-ALL, both at limiting dilution. As the strongest evidence for a rare, phenotypically immature leukaemia stem cell population in B-ALL came from experiments with Philadelphia chromosome-positive ALL (Castor et al, 2005; Cobaleda et al, 2000; Hotfilder et al, 2005), we primarily focused on patients, which were either *BCR-ABL1*-positive (five patients) or demonstrated a *BCR-ABL1*-like expression signature (two patients; Den Boer et al, 2009). To test for broader applicability to other types of B-ALL, we also included two patients with infant ALL/t(4;11) and three patients with no known cytogenetic risk factors.

Two of the primary samples engrafted only at cell doses of 5 × 10^4^ to 1 × 10^6^ cells, while the remaining nine primary leukaemias initiated the leukaemia with as few as 100–1000 cells (Table 2), with frequencies for leukaemia-propagating cells ranging from 1:40 to 1:2900. Following passage through the mice, the frequency of LPCs was even higher (1:10) and all primograft specimens engrafted with as few as 10 transplanted cells. These are much higher frequencies than reported in previous studies, where less immunodeficient SCID or NOD/SCID mice engrafted with 10,000–100,000 cells (Castor et al, 2005; Cobaleda et al, 2000; Cox et al, 2004; Hong et al, 2008; le Viseur et al, 2008). This study confirms that in many B-ALL patients, the frequency of blasts that are able to contribute to and re-grow the leukaemia is likely to be high and that the ability for clonal expansion may not be limited to a rare stem-like population.

**Table 2:** Leukaemic engraftment at limiting dilution and quantification of LPC frequencies using unsorted B-ALL blasts and blasts sorted for expression levels of CD10, CD20 and CD34

Patient ID	Transplant	Transplanted blast population	Number of engrafted mice/number of transplanted mice injected with			Frequency of leukaemia-propagating cells estimate (confidence intervals)
				
			1000 cells	100 cells	10 cells	
Primary transplants						
EMCR1	Primary	Unsorted	4/4	5/5	0/5	1:40 (1:16–112)
EMCR2	Primary	Unsorted	2/3	0/5	0/5	1:1200 (1:300–4700)
L858	Primary	Unsorted	4/5	2/5	0/5	1:460 (1:180–1200)
L876	Primary	Unsorted	4/4	4/5	0/5	1:80 (1:30–210)
2003	Primary	Unsorted	1/3	0/4	0/5	1:2900 (1:420–20,000)
4917	Primary	Unsorted	1/3	0/4	0/5	1:2900 (1:420–20,000)
L4967	Primary	Unsorted	4/4	5/5	0/5	1:40 (1:16–110)
L49120	Primary	Unsorted	1/4	1/5	0/5	1:1900 (1:540–6600)
L49101	Primary	Unsorted	2/4	0/4	0/4	1:950 (1:240–3700)
Secondary/tertiary transplants (primografts)						
L4951	Secondary	Unsorted	6/6	10/10	5/8	1:10 (1:4–25)
L49101	Secondary	Unsorted	4/4	5/5	4/5	1:6 (1:2–18)
L4967	Tertiary	Unsorted	3/3	5/5	3/5	1:11 (1:3–35)
CD10						
EMCR1	Secondary	CD10^high^	4/4	2/4	0/5	1:170 (1:47–590)
		CD10^low^	4/4	2/3	0/5	1:120 (1:30–460)
CD20						
EMCR1	Primary	CD20^high^	4/4^a^	–	–	Unable to calculate
		CD20^low^	4/4^a^	–	–	Unable to calculate
EMCR1	Secondary	CD20^high^	4/4	2/4	0/5	1:170 (1:47–590)
		CD20^low^	2/4	0/4	0/5	1:1700 (1:420–6700)
2510	Secondary	CD20^high^	2/4	0/4	–	1:4900 (1:1200–20,000)
		CD20^low^	4/4	1/4	–	1:780 (1:230–2700)
L4951	Secondary	CD20^high^	11/11	5/5	–	Unable to calculate
		CD20^low^	10/11	6/6	–	Unable to calculate
	Tertiary	CD20^high^	–	3/3	0/4	1:140 (1:25–740)
		CD20^low^	–	3/3	0/4	1:140 (1:25–740)
CD34						
L784	Primary	CD34^high^	3/4	0/5	0/5	1:930 (1:300–2.900)
		CD34^low^	3/4	1/5	0/6	1:660 (1:230–1900)
4540a	Primary	CD34^high^	2/5	3/5	0/4	1:840 (1:300–2.300)
		CD34^low^	1/5	0/5	0/5	1:5.000 (1:710–36.000)
EMCR1	Secondary	CD34^high^	4/4	1/5	3/5	1:120 (1:40–360)
		CD34^low^	4/4	2/5	1/5	1:140 (1:46–450)
L4951	Tertiary	CD34^high^	3/3	5/5	5/6	1:6 (1:2–15)
		CD34^low^	4/4	5/5	4/5	1:6 (1:2–1:18)

a Primary cells from patient EMCR1 were transplanted at a cell dose of 1500 cells/mouse. Group sizes >5 indicate pooled data from several transplantation experiments.

These data also challenged our previously published data on the engraftment of phenotypically diverse blasts as those transplants were done at high cell dose rather than at limiting dilution and in retrospect, we therefore cannot exclude that few contaminating blasts rather than the main purified population engrafted the mice. We, therefore, interrogated the frequency of leukaemia-propagating cells within the different subpopulations. Overall, sorted populations from five different patients (4 *BCR-ABL1*-positive or *BCR-ABL1*-like; 1 with no known cytogenetic risk factors) were transplanted at limiting dilution into mice (Table 2). For instance, primografted blasts from the *BCR-ABL1*-like patient EMCR1 were sorted for either CD10, CD20 or CD34 expression. All populations engrafted with an estimated propagating cell frequency of 1:120 to 1:170 (confidence intervals ranging from 1:30 to 1:590). Counterintuitively, more immature CD20^low^-expressing cells from this patient appeared to engraft less well with an estimated propagating cell frequency of 1:1700 (confidence intervals 1:420–1:6700). However, only two mice engrafted in this particular experiment and the confidence intervals overlap with the respective results for the more mature CD20^high^ blasts. Importantly, there was no correlation between propagating cell activity and CD20 status in three independent experiments with primografted cells from two additional patients. Similarly, no difference in the frequency of leukaemia-propagating cells was detected between CD34^high^ and CD34^low^ population from three out of four patients examined.

To exclude the possibility that these findings were an artefact of clonal selection or adaptation to the xeno-environment resulting from passage through the mice, we also assayed primary blasts sorted for CD20 or CD34 expression and transplanted at low cell dose. Again, all populations engrafted. Both CD20^high^ and CD20^low^ primary cells from the previously described patient, EMCR1, engrafted at a cell dose of 1500 cells confirming that there was no difference in the leukaemia-propagating potential of either population. Similarly, CD34^high^ and CD34^low^ primary cells from dic(9;20)(p13;q11)-positive patient L784 engrafted at similar frequency (1:660 to 1:930; confidence intervals ranging from 1:230–1:2900). In only one primary sample did the LPC frequency appear to be slightly higher in the phenotypically more immature CD34^high^ fraction (1:840, confidence intervals 1:300–1:2300, as compared with 1:5000, confidence intervals 1:710–1:36,000). However, the results for the CD34^low^ fraction in this experiment are based on only a single engrafted mouse and confidence intervals overlap. Although we cannot exclude the presence of a stem cell hierarchy in individual B-ALL patients when considering the variablity of the *in vivo* mouse model, our results demonstrate a comparable frequency of leukaemia-propagating cells in all populations tested.

**Figure 4:**
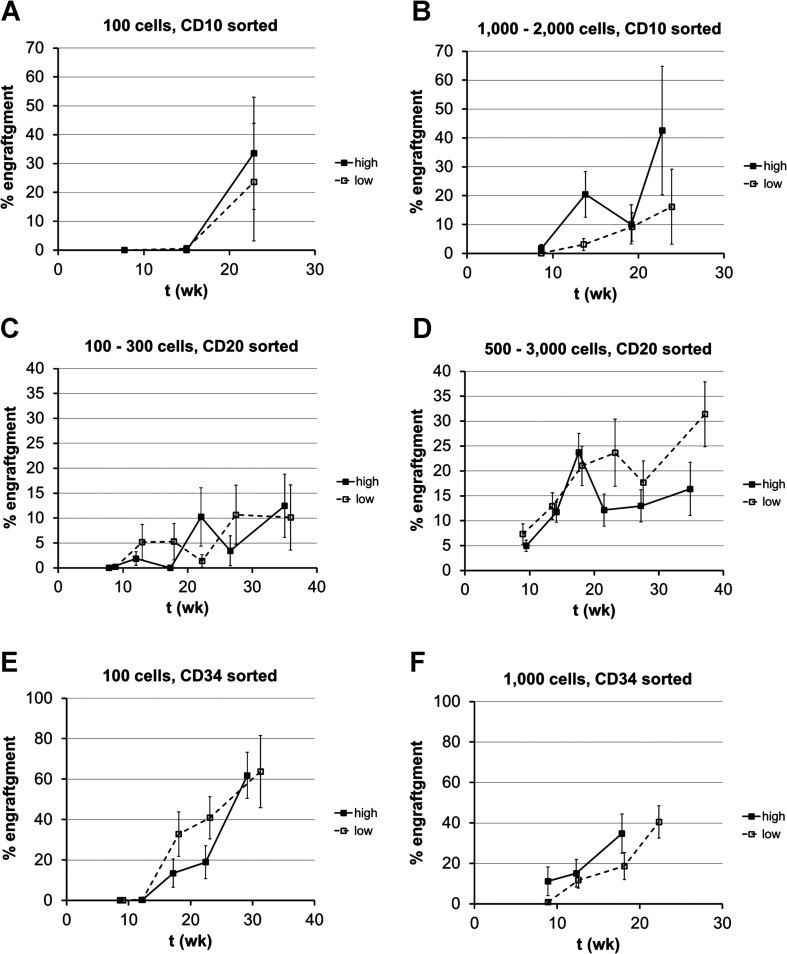
Engraftment kinetics of purified B-ALL subpopulation as assessed by sequential bone marrow punctures Mice from all limiting dilution experiments were pooled, as the number of mice in each individual transplantation experiment was too low for the analysis. Data are mean percentage of engraftment with bars showing standard error of the mean. **A,B.** Engraftment kinetics of CD10^high^ and CD10^low^ cells after transplantation of 100 (*n* = 4) or 1000–2000 blasts (*n* ≥ 4).**C,D.** Engraftment kinetics of CD20^high^ and CD20^low^ cells after transplantation of 100–300 (*n* ≥ 8) or 500–3000 blasts (*n* ≥ 21).**E,F.** Engraftment kinetics of CD34^high^ and CD34^low^ cells after transplantation of 100 (*n* ≥ 6, last points *n* = 2 for CD34^high^ and *n* = 3 for CD34^low^) or 1000 blasts (*n* ≥ 9).

Next, we tested the engraftment kinetics of the different subpopulations. To this end, we determined the leukaemic infiltration produced by CD10-, CD20- and CD34-sorted blasts over time using sequential bone marrow punctures. In order to achieve sufficient numbers of mice at each time point, all engrafted mice transplanted with either 100–300 cells or 500–3000 cells were pooled for this analysis. Each of the population engrafted with similar kinetics (Fig 4).

Since it was unclear whether more ‘mature’ immunophenotypes also have long-term self-renewal potential, we performed serial transplantations with CD20^high^ and CD34^low^ leukaemic blasts. In a proof-of-concept experiment, we were able to maintain the leukaemia with ‘mature’ CD20^high^ and CD34^low^ blasts over at least five serial transplantations (Supporting Information Fig S5). Ten thousand primary cells from *BCR-ABL1*-positive patient L4951, sorted for high CD20 expression, established the disease in primary mice. One thousand unsorted blasts from the primary mice re-initiated the leukaemia in secondary mice. One hundred blasts sorted for low expression of CD34 transferred the leukaemia onto tertiary mice and unsorted blasts from these tertiary mice have now been passaged through two more generations of mice, clearly demonstrating long-term self-renewal of these ‘mature’ B-ALL blasts. In an additional experiment, 2 × 10^4^ CD20^high^ blasts engrafted secondary mice and 2 × 10^3^ CD20^high^ cells purified from the secondary mice engrafted tertiary mice.

In summary, here we provide quantitative data on the frequency and engraftment kinetics of leukaemia-propagating cells in a range of phenotypically diverse blast cell populations from both primary and primografted samples, demonstrating that each of the human populations reads out in this NSG mouse assay with similar frequencies of leukaemia-propagating cells, similar engraftment kinetics and with long-term engraftment potential.

## DISCUSSION

Different models have been applied to explain the heterogeneity within a given tumour with respect to the ability of individual tumour cells to maintain and re-establish the disease (Bomken et al, 2010; Magee et al, 2012). The hierarchical stem cell model predicts that a tumour is maintained by a distinct, immature and typically rare cancer-propagating stem cell. The balance between self-renewal and differentiation is tightly regulated and loss of ‘stemness’ is imposed on the progeny by progressive epigenetic silencing of self-renewal genes (Broske et al, 2009). A hierarchical cancer stem cell model was first shown to apply to AML (Bonnet & Dick, 1997; Lapidot et al, 1994) and since then has been used to describe and identify cancer stem cells in a wide range of malignancies (Dick, 2008). However, the question has been raised of whether the hierarchical stem cell model applies to all cancer types (Kelly et al, 2007) and in a murine BCR-ABL-driven ALL model nearly every blast had been shown to have leukaemia-propagating potential (Williams et al, 2007). Studies with human melanoma cells also demonstrated that as many as one in four cells, irrespective of their phenotype, possess the ability to propagate the tumour (Quintana et al, 2008, 2010). This is consistent with the alternative, stochastic stem cell model in which the ability to self-renew represents a functional cellular phenotype. Execution of the ‘stemness’ programme is dependent on endogenous and exogenous (micro-environmental) signals, but the potential is present in any tumour cell.

In our previous experiments (le Viseur et al, 2008), we had demonstrated leukaemia-propagating activity in a wide range of phenotypically diverse blast populations, questioning the applicability of the hierarchical stem cell model for human B lineage acute lymphoblastic leukaemia. Although these data contradicted a number of studies investigating the phenotype of leukaemia stem cells in B-ALL (Cobaleda et al, 2000; Cox et al, 2004, 2009; Hong et al, 2008), more recent studies using improved mouse models (see discussion below) reached very similar conclusions (Diamanti et al, 2012; Kong et al, 2008; Morisot et al, 2010).

The differences between our model, suggesting a lack of a leukaemia stem cell hierarchy, and previous studies suggesting the presence of a distinct leukaemic stem cell population (Cobaleda et al, 2000; Cox et al, 2004, 2009; Hong et al, 2008) are most likely due to differences in the mouse models. Taussig and co-workers demonstrated that even in severely immunodeficient mice, antibody-coated human cells can be cleared by the residual innate immune system (Taussig et al, 2008). Other modifications to the xenograft model, such as the use of different mouse strains (McDermott et al, 2010) and the route of transplantation (Taussig et al, 2008), will also affect which cells are able to read out as stem cells and failure to engraft may have explanations other than lack of stemness (Vormoor, 2009).

Although an increasing body of data has been published, which supports our model, key questions remained unanswered. Our previous studies were biased towards high-risk infant ALL, allowing the possibility that our model may not be widely applicable to other types of ALL. More importantly, our xenotransplants primarily used primograft samples and, based on the experience in xenografted T-ALL (Chiu et al, 2010), there was concern that the phenotype of cells able to propagate the leukaemia may be unstable and change following passage through the mice. To address these concerns, we have now used primary in addition to primograft samples, samples from patients reflecting a wider range of acute B lymphoblastic leukaemia (B-ALL) subtypes (Table 1) and purified blasts based on the expression of additional B-cell differentiation markers. Our experiments demonstrate that this model is indeed more widely applicable to B-ALL, including primary patient samples, and we show that cells with leukaemia-propagating potential are present in phenotypically diverse blast cell populations, specifically in CD10^low/high^, CD20^low/high^ and CD34^low/high^ cells.

One of the key limitations of previous experiments was the lack of quantitative data on the frequency of LPCs in unseparated primary blasts and in the different blast populations. Our model suggests that leukaemia-propagating activity is not limited to a phenotypically rare cell population but may be quite frequent. Indeed, we now show that the frequency of B-ALL blasts able to initiate human leukaemia in immunodeficient NSG mice is high, with many primary leukaemias able to engraft with as few as 100 cells. Propagating cell frequencies increase further after passage through the mice, most likely due to selection of clones better adapted to the murine microenvironment (Clappier et al, 2011). Growth and growth kinetics of B-ALL in immunodeficient mice have indeed been correlated with expression of apoptosis resistance genes (Meyer et al, 2011). This suggests that in patients—where there is no xenograft barrier and no *ex vivo* manipulation—the frequency of blasts that are able to contribute to and re-grow the leukaemia is likely to be higher than that assessed in a xeno-environment. Similar studies reporting a high frequency of leukaemia-propagating cells in five primary samples (Morisot et al, 2010) and in primografts (Schmitz et al, 2011) have also recently been described by others.

Importantly, we demonstrate here that propagating cell frequencies and engraftment kinetics are similar across a wide range of populations, including immunophenotypically more mature blasts, at least within a cohort biased towards Philadelphia-chromosome-positive ALL. These experiments are consistent with the stochastic stem cell model in these patients. Together with the demonstration of a high frequency of leukaemia-propagating cells in non-high-risk patients (L784, L858 and 4917) and the comparable engraftment of different lymphoid subpopulations from a wide range of patients, these data suggest that the lack of a stem cell hierarchy may be a more universal feature of B-ALL.

**Figure 5:**
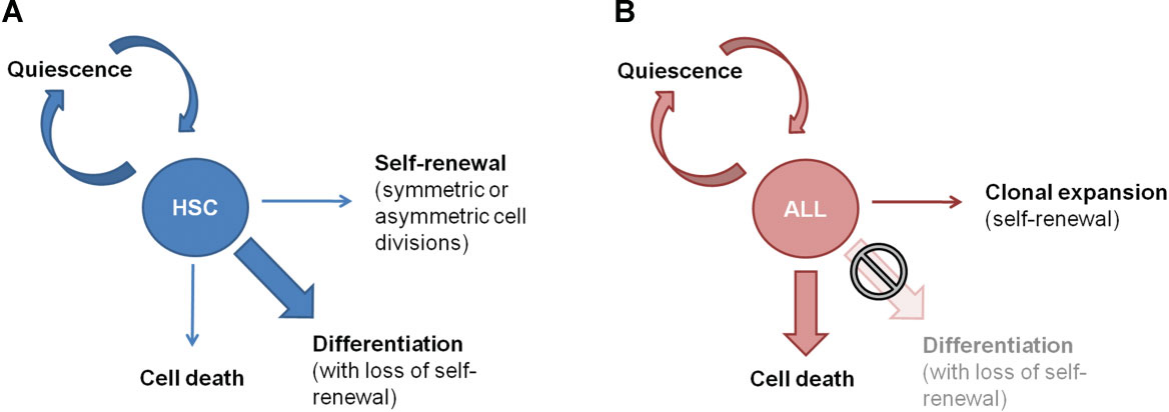
Models for regulation of self-renewal, proliferation and differentiation in normal haematopoietic stem cells and acute myeloid leukaemia as compared with acute lymphoblastic leukaemia In normal haematopoietic stem cells and AML, self-renewal is tightly regulated by a differentiation-dependent loss of self-renewal.In B-ALL, clonal expansion is tightly regulated by the dependence of positive survival and proliferation signals. Without those lymphoid blasts undergo apoptosis.

Our xenotransplantation data suggested that blasts sorted for different surface markers should express key components of cellular programmes that are essential for a high, if not unlimited, proliferative potential. The candidate gene we chose to test this hypothesis was *TERT,* which controls self-renewal of normal and cancer stem cells, and whose expression is maintained by key leukaemic fusion oncogenes supporting its essential role in leukaemic propagation (Gessner et al, 2010). As predicted, quantitative RT-PCR analysis revealed that there were no differences in *TERT* expression between CD34^high^ and CD34^low^ leukaemic blasts. This is similar to normal B-cell development: in line with the high proliferative potential, lymphocytes are the only cellular compartment in humans in which *TERT* expression is maintained even during the late stages of differentiation and can be up-regulated with activation and proliferation (Liu et al, 1999; Lobetti-Bodoni et al, 2010; Son et al, 2003). Notably, none of gene expression signatures generated from AML or normal cells with self-renewal potential (Eppert et al, 2011; Gentles et al, 2010; Kim et al, 2009) distinguished CD34^high^ from CD34^low^ blasts in B-ALL, indicating their similarity in relation to expression of candidate stem cell genes. These expression data are consistent with our model that, unlike the myeloid lineage, B-ALL shows no dissociation between self-renewal and differentiation (Fig 5). Notably, one of the early pioneers of stem cell biology, the late Dr Ernest McCulloch, stated that ‘a minimum conclusion to be reached from the comparison of myelopoiesis and lymphopoiesis is that a firm linkage between differentiation and loss of proliferative capacity is not a general characteristic of hemopoiesis’ (McCulloch, 1983). Normal mature lymphoid cells maintain their ability to clonally expand—a characteristic, which is a core element of the lymphocyte response to re-exposure to antigen. Lymphoid cells can therefore be considered unipotent stem cells and we show here that this may also apply to malignant blasts in B lineage acute leukaemia.

A model of clonal evolution has recently been proposed for B-ALL (Anderson et al, 2011; Notta et al, 2011). Although both the hierarchical and the stochastic stem cell model are compatible with clonal evolution, the high frequency of phenotypically diverse leukaemia-propagating cells as described here (stochastic model) will more easily facilitate clonal evolution and the generation of clonal complexity.

In summary, our experiments demonstrate that, consistent with the stochastic model, many lymphoid blasts with diverse immunophenotypes can clonally expand and propagate the leukaemia. Unravelling the mechanisms of clonal expansion will provide novel therapeutic targets that may not only be exploited for the treatment of B-ALL, but may also be applicable to other lymphoid malignancies and autoimmunity.

## MATERIALS AND METHODS

### Patient samples

Bone marrow and peripheral blood samples were collected as part of initial diagnostic investigations. Written informed consent was obtained from patients and/or legal guardians according to protocols approved by the corresponding review boards. Patient samples from the UK that were stored before September 2006 were exempt from specific consent for research by the Human Tissue Act. Anonymized umbilical cord blood specimens, stored with consent for research, were obtained from the Newcastle Umbilical Cord Blood Bank, Newcastle University.

### Flow cytometry

Mononuclear cells from thawed diagnostic bone marrow or peripheral blood samples and freshly recovered bone marrow from transplanted mice were analysed by 5-colour flow cytometry. Samples were collected into RPMI 1640 medium containing 10% FCS and re-suspended in phosphate-buffered saline (PBS) containing 0.2% bovine serum albumin (PBSA). Diagnostic samples were incubated with saturating amounts of monoclonal antibodies (BD Biosciences, Oxford, UK) to human surface proteins of interest in a total volume of 200 µl for 20 min at room temperature in the dark. Antibodies used were: anti-CD10 FITC (clone HI10a); anti-CD19 PE (clone SJ25C1); anti-CD20 PerCP-Cy5.5 (clone L27); anti-CD38 PE-Cy7 (clone HB-7); anti-CD34 APC (clone 8G12). For bone marrow samples harvested from mice, anti-CD38 PE-Cy7 was exchanged for murine anti-TER119 PE-Cy7 and murine anti-CD45 PE-Cy7 (clone 30-F11) to exclude contaminating murine cells. Stained samples were washed twice and resuspended in 400 µl PBSA for analysis. All flow cytometric analyses were performed on a FACSCanto II flow cytometer (BD Biosciences) using FACSDiva analysis software v6.1. Cell sorting was performed on a FACSVantage SE cell sorter (BD Biosciences) using CellQuest Pro software. Staining for cell sorting was performed as described above using anti-CD19 PE, anti-CD10 FITC, anti-CD20 FITC (clone L27) or anti-CD34 FITC (clone 8G12). Mean purities of the sorted populations were: CD10^high^—98%, CD10^low^—99%, CD20^high^—95%, CD20^low^—99%, CD34^high^—97% and CD34^low^—99%.

### Animal model

All transplantation experiments were performed using NSG mice (NOD.Cg-Prkdcscid Il2rgtm1Wjl/SzJ) mice; Jackson Laboratories, Bar Harbor, ME, USA) (Shultz et al, 2005). All handling and experimental manipulation of these animals was performed in a laminar flow hood using aseptic techniques. Mice used for transplantation were at least 6 weeks of age (body weight 20–30 g). Prior to transplantation, mice were anaesthetized with 2.5–5% isoflurane in 1.5 L/min of oxygen, followed by subcutaneous administration of Carprofen (Rimadyl, Pfizer, UK) 5 mg/kg. The femur was punctured with a 27G or 30G needle (BD Biosciences) followed by injection of up to 30 µl of cell suspension using a 0.5 ml insulin syringe (30 G). To obtain bone marrow aspirates, the femur was punctured with a 25 G needle, followed by aspiration using a fresh 25 G needle into a syringe filled with 400 µl of RPMI 1640 medium containing 20% FCS. The health status of the mice was checked clinically on a daily basis and all mice were weighed on a weekly basis. As soon as mice developed clinical signs of leukaemia development or had a weight loss of more than 20%, they were humanely killed. Due to the normal variability of the xenograft assay and depending on the number of viable leukaemic blasts available we aimed for group sizes of 3–5 animals per purified population, patient and cell dose. All animal experiments were approved under the UK Home Office licence (PPL 60/3846).

### Calculation of propagating cell frequencies

LPC frequencies were calculated using the open access programme ELDA, http://bioinf.wehi.edu.au/software/elda/ (Hu & Smyth, 2009). For the purpose of this study, the analysis is based on the assumption that the frequency of propagating cells in the different inocula follows the Poisson distribution with one cell being sufficient to re-grow the leukaemia (Poisson single hit model).

### *TERT* expression in sorted blast populations and normal umbilical cord blood

Leukaemic CD34^low^ and CD34^high^ populations were sorted as described. Thawed umbilical cord blood was washed in 500 ml RPMI 1640 supplemented with 10% FCS and resuspended in 50 ml DNase buffer (0.5 mM MgCl_2_, 1 mM CaCl_2_, 0.5% w/v PBSA). Cells were incubated with 5000 U DNase I (Sigma) for 45 min at room temperature. Washed cells were labelled with saturating amounts of FcR blocking reagent and MACS anti-CD34 microbeads (Miltenyi Biotec, Bergisch Gladbach, Germany). CD34^high^ cells were purified by serial passage down two LS positive selection columns according to the manufacturer's instructions. Unlabeled flow-through cells were collected to provide the CD34 negative population. RNA was isolated from sorted populations using an RNeasy kit according to the manufacturer's instructions (Qiagen, Hilden, Germany).

**The paper explained**

PROBLEM:

The hierarchical stem cell model predicts that a tumour is maintained by a distinct cancer propagating stem cell, which therefore represents the principal target of any curative treatment.

RESULTS:

Here, we show that propagating cells in acute lymphoblastic leukaemia cannot be enriched by purification based on a range of B-cell maturation markers. All purified populations engraft with similar and high frequency and with similar kinetics and furthermore each population is able to replenish the others. These data are consistent with the lack of a stem cell hierarchy in acute B lymphoblastic leukaemia and, in principle, all blasts may have the ability to propagate the leukaemia.

IMPACT:

Unravelling the mechanisms, which regulate and drive clonal expansion in malignant lymphoid blasts, will provide new therapeutic targets that are likely to be relevant not only for rare childhood leukaemia but may also apply to other lymphoid malignancies and autoimmunity.

RNA was reverse transcribed using RevertAid™ H Minus First Strand cDNA Synthesis Kit (Fermentas). The qRT PCR reaction was performed in 10 µl on a 384-well plate using 20% v/v cDNA, 50% v/v 2× Platinum SYBR QPCR Supermix with ROX (Invitrogen) and 0.3 µM primers: TBP Fw: 5′—CCT AAA GAC CAT TGC ACT TCG T—3′, Re: 5′—GTT CGT GGC TCT CTT ATC CTC A—3′; CD34 Fw: 5′—AAA GCA CCA ATC TGA CCT GAA A—3′, Re: 5′—CGA GGT GAC CAG TGC AAT CA—3′; TERT Fw: 5′—GGA GAA CAA GCT GTT TGC GG—3′, Re: 5′—AGG TTT TCG CGT GGG TGA G—3′. Reaction conditions were: 2 min at 50°C, 10 min at 95°C followed by 40 cycles of 15 s at 95°C, 1 min at 60°C followed by 15 s at 95°C, 15 s at 60°C and 15 s at 95°C. Quantitative analysis was performed using a 7900HT Sequence Detection System (Applied Biosystems). Data analysis was performed using SDS 2.2 software (Applied Biosystems) by Δ*C*_t_ comparison, using *GAPDH* as a reference transcript.

### Expression profiling of CD34^low^ and CD34^high^ blasts

Diagnostic patient bone marrow was sorted for CD34^high/low^ expression as previously described (le Viseur et al, 2008). Isolated RNA was taken forward for expression microarray analysis using the Affymetrix Genechip HG-U133 Plus 2.0 array (GeneService Ltd.). Data are available at ArrayExpress (http://www.ebi.ac.uk/microarray-as/aer/) under the accession number E-MEXP-1522.

CEL files from Affymetrix HGU133plus2 profiles representing the different haematopoietic subpopulations were taken from GEO Series GSE8023 (Krejci et al, 2008), GSE17186 (Suryani et al, 2010), GSE19599 (Andersson et al, 2007), GSE24006 (Gentles et al, 2010) and normalized along with profiles from CD34 sorted cells from patients L787, L812 and L776 using gc Robust Multiarray Average (gcRMA). Two sets of genes were selected. The first reflected hematopoietic ‘self-renewal’ genes (a 57 gene 231 probe signature taken from (Kim et al, 2009) and an AML LSC signature from (Eppert et al, 2011). The second consisted of 33 probes, highly differentially expressed between CD34^high^ and CD34^low^ subpopulations showing greater than threefold difference in expression and greater than 100-fold difference in absolute intensity in each patient. PCA was performed using R or TMEV package (http://www.tm4.org/mev/) where appropriate. Heatmaps were generated using Genepattern (http://www.broadinstitute.org/cancer/software/genepattern/). GSEA was performed using the stand alone application http://www.broadinstitute.org/gsea/ using MSigDB C2v3 geneset database with a custom geneset added containing 204 genes at least threefold mean differentially expressed between pro-B cells and mature B-cell expression profiles as found in GSE19599.

## For more information

Childhood acute lymphoblastic leukaemia

http://www.cclg.org.uk/childhood-cancer/leukaemias

http://cancerhelp.cancerresearchuk.org/type/all

http://leukaemialymphomaresearch.org.uk/information/childhood-leukaemia/acute-lymphoblastic-leukaemia

## Supplementary material

Detailed facts of importance to specialist readers are published as ”Supporting Information”. Such documents are peer-reviewed, but not copy-edited or typeset. They are made available as submitted by the authors.

## References

[b1] Al-Hajj M, Wicha MS, Benito-Hernandez A, Morrison SJ, Clarke MF (2003). Prospective identification of tumorigenic breast cancer cells. Proc Natl Acad Sci USA.

[b2] Anderson K, Lutz C, van Delft FW, Bateman CM, Guo Y, Colman SM, Kempski H, Moorman AV, Titley I, Swansbury J (2011). Genetic variegation of clonal architecture and propagating cells in leukaemia. Nature.

[b3] Andersson A, Ritz C, Lindgren D, Eden P, Lassen C, Heldrup J, Olofsson T, Rade J, Fontes M, Porwit-Macdonald A (2007). Microarray-based classification of a consecutive series of 121 childhood acute leukemias: prediction of leukemic and genetic subtype as well as of minimal residual disease status. Leukemia.

[b4] Bomken S, Fiser K, Heidenreich O, Vormoor J (2010). Understanding the cancer stem cell. Br J Cancer.

[b5] Bonnet D, Dick JE (1997). Human acute myeloid leukemia is organized as a hierarchy that originates from a primitive hematopoietic cell. Nat Med.

[b6] Broske AM, Vockentanz L, Kharazi S, Huska MR, Mancini E, Scheller M, Kuhl C, Enns A, Prinz M, Jaenisch R (2009). DNA methylation protects hematopoietic stem cell multipotency from myeloerythroid restriction. Nat Genet.

[b7] Castor A, Nilsson L, Astrand-Grundstrom I, Buitenhuis M, Ramirez C, Anderson K, Strombeck B, Garwicz S, Bekassy AN, Schmiegelow K (2005). Distinct patterns of hematopoietic stem cell involvement in acute lymphoblastic leukemia. Nat Med.

[b8] Chiu PP, Jiang H, Dick JE (2010). Leukemia-initiating cells in human T-lymphoblastic leukemia exhibit glucocorticoid resistance. Blood.

[b9] Clappier E, Gerby B, Sigaux F, Delord M, Touzri F, Hernandez L, Ballerini P, Baruchel A, Pflumio F, Soulier J (2011). Clonal selection in xenografted human T cell acute lymphoblastic leukemia recapitulates gain of malignancy at relapse. J Exp Med.

[b10] Cobaleda C, Gutierrez-Cianca N, Perez-Losada J, Flores T, Garcia-Sanz R, Gonzalez M, Sanchez-Garcia I (2000). A primitive hematopoietic cell is the target for the leukemic transformation in human philadelphia-positive acute lymphoblastic leukemia. Blood.

[b11] Cox CV, Evely RS, Oakhill A, Pamphilon DH, Goulden NJ, Blair A (2004). Characterization of acute lymphoblastic leukemia progenitor cells. Blood.

[b12] Cox CV, Diamanti P, Evely RS, Kearns PR, Blair A (2009). Expression of CD133 on leukemia-initiating cells in childhood ALL. Blood.

[b13] Den Boer ML, van Slegtenhorst M, De Menezes RX, Cheok MH, Buijs-Gladdines JG, Peters ST, Van Zutven LJ, Beverloo HB, Van der Spek PJ, Escherich G (2009). A subtype of childhood acute lymphoblastic leukaemia with poor treatment outcome: a genome-wide classification study. Lancet Oncol.

[b14] Diamanti P, Cox CV, Blair A (2012). Comparison of childhood leukemia initiating cell populations in NOD/SCID and NSG mice. Leukemia.

[b15] Dick JE (2008). Stem cell concepts renew cancer research. Blood.

[b16] Dieter SM, Ball CR, Hoffmann CM, Nowrouzi A, Herbst F, Zavidij O, Abel U, Arens A, Weichert W, Brand K (2011). Distinct types of tumor-initiating cells form human colon cancer tumors and metastases. Cell Stem Cell.

[b17] Eppert K, Takenaka K, Lechman ER, Waldron L, Nilsson B, van Galen P, Metzeler KH, Poeppl A, Ling V, Beyene J (2011). Stem cell gene expression programs influence clinical outcome in human leukemia. Nat Med.

[b18] Eramo A, Lotti F, Sette G, Pilozzi E, Biffoni M, Di Virgilio A, Conticello C, Ruco L, Peschle C, De Maria R (2008). Identification and expansion of the tumorigenic lung cancer stem cell population. Cell Death Differ.

[b19] Gentles AJ, Plevritis SK, Majeti R, Alizadeh AA (2010). Association of a leukemic stem cell gene expression signature with clinical outcomes in acute myeloid leukemia. JAMA.

[b20] Gessner A, Thomas M, Castro PG, Buchler L, Scholz A, Brummendorf TH, Soria NM, Vormoor J, Greil J, Heidenreich O (2010). Leukemic fusion genes MLL/AF4 and AML1/MTG8 support leukemic self-renewal by controlling expression of the telomerase subunit TERT. Leukemia.

[b21] Goardon N, Marchi E, Atzberger A, Quek L, Schuh A, Soneji S, Woll P, Mead A, Alford KA, Rout R (2011). Coexistence of LMPP-like and GMP-like leukemia stem cells in acute myeloid leukemia. Cancer Cell.

[b22] Hemmati HD, Nakano I, Lazareff JA, Masterman-Smith M, Geschwind DH, Bronner-Fraser M, Kornblum HI (2003). Cancerous stem cells can arise from pediatric brain tumors. Proc Natl Acad Sci USA.

[b23] Ho MM, Ng AV, Lam S, Hung JY (2007). Side population in human lung cancer cell lines and tumors is enriched with stem-like cancer cells. Cancer Res.

[b24] Hong D, Gupta R, Ancliff P, Atzberger A, Brown J, Soneji S, Green J, Colman S, Piacibello W, Buckle V (2008). Initiating and cancer-propagating cells in TEL-AML1-associated childhood leukemia. Science (New York).

[b25] Hope KJ, Jin L, Dick JE (2004). Acute myeloid leukemia originates from a hierarchy of leukemic stem cell classes that differ in self-renewal capacity. Nat Immunol.

[b26] Hotfilder M, Rottgers S, Rosemann A, Schrauder A, Schrappe M, Pieters R, Jurgens H, Harbott J, Vormoor J (2005). Leukemic stem cells in childhood high-risk ALL/t(9;22) and t(4;11) are present in primitive lymphoid-restricted CD34+CD19-cells. Cancer Res.

[b27] Hu Y, Smyth GK (2009). ELDA: extreme limiting dilution analysis for comparing depleted and enriched populations in stem cell and other assays. J Immunol Methods.

[b28] Irving J, Jesson J, Virgo P, Case M, Minto L, Eyre L, Noel N, Johansson U, Macey M, Knotts L (2009). Establishment and validation of a standard protocol for the detection of minimal residual disease in B lineage childhood acute lymphoblastic leukemia by flow cytometry in a multi-center setting. Haematologica.

[b29] Jamieson CH, Ailles LE, Dylla SJ, Muijtjens M, Jones C, Zehnder JL, Gotlib J, Li K, Manz MG, Keating A (2004). Granulocyte-macrophage progenitors as candidate leukemic stem cells in blast-crisis CML. N Engl J Med.

[b30] Kelly PN, Dakic A, Adams JM, Nutt SL, Strasser A (2007). Tumor growth need not be driven by rare cancer stem cells. Science (New York, NY).

[b31] Kim YC, Wu Q, Chen J, Xuan Z, Jung YC, Zhang MQ, Rowley JD, Wang SM (2009). The transcriptome of human CD34+ hematopoietic stem-progenitor cells. Proc Natl Acad Sci USA.

[b32] Kong Y, Yoshida S, Saito Y, Doi T, Nagatoshi Y, Fukata M, Saito N, Yang SM, Iwamoto C, Okamura J (2008). CD34+CD38+CD19+ as well as CD34+CD38-CD19+ cells are leukemia-initiating cells with self-renewal capacity in human B-precursor ALL. Leukemia.

[b33] Krejci O, Wunderlich M, Geiger H, Chou FS, Schleimer D, Jansen M, Andreassen PR, Mulloy JC (2008). p53 signaling in response to increased DNA damage sensitizes AML1-ETO cells to stress-induced death. Blood.

[b34] Lapidot T, Sirard C, Vormoor J, Murdoch B, Hoang T, Caceres-Cortes J, Minden M, Paterson B, Caligiuri MA, Dick JE (1994). A cell initiating human acute myeloid leukaemia after transplantation into SCID mice. Nature.

[b35] le Viseur C, Hotfilder M, Bomken S, Wilson K, Rottgers S, Schrauder A, Rosemann A, Irving J, Stam RW, Shultz LD (2008). In childhood acute lymphoblastic leukemia, blasts at different stages of immunophenotypic maturation have stem cell properties. Cancer Cell.

[b36] Liu K, Schoonmaker MM, Levine BL, June CH, Hodes RJ, Weng NP (1999). Constitutive and regulated expression of telomerase reverse transcriptase (hTERT) in human lymphocytes. Proc Natl Acad Sci USA.

[b37] Lobetti-Bodoni C, Bernocco E, Genuardi E, Boccadoro M, Ladetto M (2010). Telomeres and telomerase in normal and malignant B-cells. Hematol Oncol.

[b38] Magee JA, Piskounova E, Morrison SJ (2012). Cancer stem cells: impact, heterogeneity, and uncertainty. Cancer Cell.

[b39] McCulloch EA (1983). Stem cells in normal and leukemic hemopoiesis (Henry Stratton Lecture, 1982). Blood.

[b40] McDermott SP, Eppert K, Lechman ER, Doedens M, Dick JE (2010). Comparison of human cord blood engraftment between immunocompromised mouse strains. Blood.

[b41] Meyer LH, Eckhoff SM, Queudeville M, Kraus JM, Giordan M, Stursberg J, Zangrando A, Vendramini E, Moricke A, Zimmermann M (2011). Early relapse in all is identified by time to leukemia in NOD/SCID mice and is characterized by a gene signature involving survival pathways. Cancer Cell.

[b42] Morisot S, Wayne AS, Bohana-Kashtan O, Kaplan IM, Gocke CD, Hildreth R, Stetler-Stevenson M, Walker RL, Davis S, Meltzer PS (2010). High frequencies of leukemia stem cells in poor-outcome childhood precursor-B acute lymphoblastic leukemias. Leukemia.

[b43] Notta F, Mullighan CG, Wang JC, Poeppl A, Doulatov S, Phillips LA, Ma J, Minden MD, Downing JR, Dick JE (2011). Evolution of human BCR-ABL1 lymphoblastic leukaemia-initiating cells. Nature.

[b44] O'Brien CA, Pollett A, Gallinger S, Dick JE (2007). A human colon cancer cell capable of initiating tumour growth in immunodeficient mice. Nature.

[b45] Quintana E, Shackleton M, Sabel MS, Fullen DR, Johnson TM, Morrison SJ (2008). Efficient tumour formation by single human melanoma cells. Nature.

[b46] Quintana E, Shackleton M, Foster HR, Fullen DR, Sabel MS, Johnson TM, Morrison SJ (2010). Phenotypic heterogeneity among tumorigenic melanoma cells from patients that is reversible and not hierarchically organized. Cancer Cell.

[b47] Ricci-Vitiani L, Lombardi DG, Pilozzi E, Biffoni M, Todaro M, Peschle C, De Maria R (2007). Identification and expansion of human colon-cancer-initiating cells. Nature.

[b48] Schmitz M, Breithaupt P, Scheidegger N, Cario G, Bonapace L, Meissner B, Mirkowska P, Tchinda J, Niggli FK, Stanulla M (2011). Xenografts of highly resistant leukemia recapitulate the clonal composition of the leukemogenic compartment. Blood.

[b49] Shultz LD, Lyons BL, Burzenski LM, Gott B, Chen X, Chaleff S, Kotb M, Gillies SD, King M, Mangada J (2005). Human lymphoid and myeloid cell development in NOD/LtSz-scid IL2R gamma null mice engrafted with mobilized human hemopoietic stem cells. J Immunol.

[b50] Singh SK, Clarke ID, Terasaki M, Bonn VE, Hawkins C, Squire J, Dirks PB (2003). Identification of a cancer stem cell in human brain tumors. Cancer Res.

[b51] Somervaille TC, Matheny CJ, Spencer GJ, Iwasaki M, Rinn JL, Witten DM, Chang HY, Shurtleff SA, Downing JR, Cleary ML (2009). Hierarchical maintenance of MLL myeloid leukemia stem cells employs a transcriptional program shared with embryonic rather than adult stem cells. Cell Stem Cell.

[b52] Son NH, Joyce B, Hieatt A, Chrest FJ, Yanovski J, Weng NP (2003). Stable telomere length and telomerase expression from naive to memory B-lymphocyte differentiation. Mech Ageing Dev.

[b53] Stewart SA, Hahn WC, O'Connor BF, Banner EN, Lundberg AS, Modha P, Mizuno H, Brooks MW, Fleming M, Zimonjic DB, Popescu NC, Weinberg RA (2002). Telomerase contributes to tumorigenesis by a telomere length-independent mechanism. Proc Natl Acad Sci USA.

[b54] Suryani S, Fulcher DA, Santner-Nanan B, Nanan R, Wong M, Shaw PJ, Gibson J, Williams A, Tangye SG (2010). Differential expression of CD21 identifies developmentally and functionally distinct subsets of human transitional B cells. Blood.

[b55] Taussig DC, Miraki-Moud F, Anjos-Afonso F, Pearce DJ, Allen K, Ridler C, Lillington D, Oakervee H, Cavenagh J, Agrawal SG (2008). Anti-CD38 antibody-mediated clearance of human repopulating cells masks the heterogeneity of leukemia-initiating cells. Blood.

[b56] Taussig DC, Vargaftig J, Miraki-Moud F, Griessinger E, Sharrock K, Luke T, Lillington D, Oakervee H, Cavenagh J, Agrawal SG (2010). Leukemia-initiating cells from some acute myeloid leukemia patients with mutated nucleophosmin reside in the CD34(−) fraction. Blood.

[b57] Till JE, McCulloch EA (1961). A direct measurement of the radiation sensitivity of normal mouse bone marrow cells. Radiat Res.

[b58] van Zelm MC, van der Burg M, de Ridder D, Barendregt BH, de Haas EF, Reinders MJ, Lankester AC, Revesz T, Staal FJ, van Dongen JJ (2005). Ig gene rearrangement steps are initiated in early human precursor B cell subsets and correlate with specific transcription factor expression. J Immunol.

[b59] Vormoor HJ (2009). Malignant stem cells in childhood acute lymphoblastic leukemia: the stem cell concept revisited. Cell Cycle.

[b60] Williams RT, den Besten W, Sherr CJ (2007). Cytokine-dependent imatinib resistance in mouse BCR-ABL+, Arf-null lymphoblastic leukemia. Genes Dev.

[b61] Zuber J, Rappaport AR, Luo W, Wang E, Chen C, Vaseva AV, Shi J, Weissmueller S, Fellmann C, Taylor MJ (2011). An integrated approach to dissecting oncogene addiction implicates a Myb-coordinated self-renewal program as essential for leukemia maintenance. Genes Dev.

