# Supplementary material for: Acute B lymphoblastic leukaemia-propagating cells are present at high frequency in diverse lymphoblast populations
Source: EMBO Mol Med. 2012 Dec 11;5(1):38–51. doi: 10.1002/emmm.201201703 (PMC3569652; doi:10.1002/emmm.201201703)
Supplement: Supplementary file 1 [file emmm0005-0038-SD1.pdf]

EMBO Molecular Medicine

Manuscript EMM-2012-01703

Acute B lymphoblastic leukaemia propagating cells are present at high frequency in diverse lymphoblast populations

Klaus Rehe, Kerrie Wilson, Simon Bomken, Daniel Williamson, Julie Irving, Monique L. den Boer, Martin Stanulla, Martin Schrappe, Andrew G. Hall, Olaf Heidenreich, and Josef Vormoor

Corresponding author: Josef Vormoor, Newcastle University

Review timeline:

Submission date:	05 July 2012
Editorial Decision:	14 August 2012
Revision received:	13 September 2012
Editorial Decision:	04 October 2012
Revision received:	15 October 2012
Accepted:	16 October 2012

Transaction Report:

(Note: With the exception of the correction of typographical or spelling errors that could be a source of ambiguity, letters and reports are not edited. The original formatting of letters and referee reports may not be reflected in this compilation.)

1st Editorial Decision 14 August 2012

Thank you for the submission of your manuscript "Acute lymphoblastic leukaemia propagating cells are present at high frequency in diverse lymphoblast populations" to EMBO Molecular Medicine and please accept my apologies for the delay. We have now received two out of three referee reports on it and given that the review process so far has been lengthy, we would prefer to make a decision now. We will send you the third report as soon as it becomes available.

You will be glad to see that the reviewers are positive about your manuscript and recommend publication of the paper pending some revisions. These are mostly minor changes that we believe you can address easily and I would therefore invite you to revise your manuscript and address the reviewers' concerns. Specifically, Reviewer #1 raises concerns about the limited dilution data and recommends to further investigate differences in leukemogenic cell frequency.

On a more editorial note, please include the section 'The paper explained' and (if you would like to) 'For more information' as detailed below. Importantly, the description of all reported data that includes statistical testing must state the name of the statistical test used to generate error bars and P values, the number (n) of independent experiments underlying each data point (not replicate measures of one sample), and the actual P value for each test (not merely 'significant' or 'P < 0.05') (please see our Instructions to Authors for more details).

Revised manuscripts should be submitted within three months of a request for revision. They will otherwise be treated as new submissions, unless arranged differently with the editor.

I look forward to seeing a revised form of your manuscript as soon as possible.

Yours sincerely,

Editor
EMBO Molecular Medicine

***** Reviewer's comments *****

Referee #1 (Comments on Novelty/Model System):

It is true that some of the content in this paper overlaps with prior papers. However I believe this paper to be the most definitive functional analysis yet. Given that this has been a controversial area, with multiple papers in high impact journals concluding based on flawed mouse models that B-ALL follows a cancer stem cell model, it is worth publishing this technically superior and more comprehensive functional analysis.

Referee #1 (Other Remarks):

Rehe et al. characterize the frequency and surface marker phenotypes of leukemia-initiating cells in B-cell acute lymphoblastic leukemia (B-ALL). This paper builds upon previously published work from the same lab (le Viseur et al., Cancer Cell 14:47). In the current study, the authors show that leukemogenic cells are present in diverse populations of B-ALL cells based upon expression of CD20, CD10 and CD34. This is true for both primary and passaged leukemia cells. Heterogeneity among leukemogenic cells was observed in both high-risk (9:22 and 11q23 mutated) and low-risk (hyperdiploid) specimens. The authors performed limit dilution experiments and concluded that B-ALL does not follow a hierarchical stem cell model. The conclusions of this paper support an emerging body of literature indicating that leukemogenic B-ALL cells are common (Diamanti et al., Leukemia, 26:376) and have diverse immunophenotypes (le Viseur et al., Cancer Cell 14:47; Kong et al., Leukemia, 22:1207; Morisot et al., Leukemia, 24:1859). The current study extends previous studies by performing limit dilution assays of multiple subpopulations of leukemia cells. It is hard to draw any conclusion from the gene expression profile data in Figures 3 and 4 of this manuscript because of our limited knowledge regarding the genes that drive clonogenicity in B-ALL and because negative data regarding gene signatures does not prove anything. The real strength in this manuscript is the extensive functional analysis, which to my knowledge is the most comprehensive yet performed in B-ALL. The use of an improved mouse model that is more permissive for engraftment by human B-ALL cells is likely part of the reason why multiple prior studies incorrectly concluded that leukemogenic cells were rare and phenotypingally distinct in B-ALL.

1. In some cases, the limit dilution data argued against a hierarchical stem cell model, but in other cases the data were consistent with a hierarchical model (Table 2). For example, in leukemia sample EMCR1, leukemogenic cells were present in the CD20high fraction at a frequency of 1:170 and in the CD20low fraction at a frequency of 1:1700 (10-fold difference). In leukemia sample 2510, leukemogenic cells were present in the CD20high fraction at a frequency of 1:4900 and in the CD20low fraction at a frequency of 1:780 (6-fold difference). It is possible that these differences are due to technical variance. It is also possible that some leukemias are organized hierarchically, but that the markers only enrich leukemogenic cells in a subset of patients. It would be best to obtain additional data for the samples in question to determine whether differences in leukemogenic cell frequency are real or a technical aberration.

2. It is not clear what the novel conclusion can be drawn from Figure 3, or how these data contribute to this manuscript. This lab has previously published microarray data comparing CD34high cells to CD34low cells (le Viseur et al., Cancer Cell 14:47). They reached the same conclusion - that decreased CD34 expression correlates with increased expression of B-cell differentiation genes, and that CD34 expression therefore distinguishes biologically different subpopulations of leukemia cells. Whether or not CD34low cells exhibit a more differentiated gene expression profile has little bearing on the relative frequency of leukemogenic cells in each subpopulation. I would recommend removing Figure 3 from this manuscript.

3. Related to point #2, the authors put too much emphasis on the idea that a hierarchical stem cell model assumes a differentiation of leukemogenic cells into non-leukemogenic progeny according to markers similar to the differentiation of normal hematopoietic cells. For example, the sentence "The inability to identify populations by marker combinations that correspond to the well defined stages of pro-B to pre-B maturation, raises the question about the relevance of the expression levels of individual surface markers in a differentiation context". This is an overly strict interpretation of the cancer stem cell model. The mutations that transform normal cells into cancer cells might also change marker expression, alter gene expression profiles, or modify hierarchies. Therefore, a cancer can follow the stem cell model and be hierarchically organized, even if the markers that distinguish leukemogenic from non-leukemogenic cells are not the same as the markers that distinguish undifferentiated from differentiated cells in the normal lineage.

4. Similarly, it is not clear what the data on TERT expression really demonstrate (Fig. 4A). TERT expression levels have never been shown to distinguish tumorigenic from non-tumorigenic cells in a cancer that follows a cancer stem cell model. The fact that TERT is expressed at similar levels in CD34high and CD34low cells does not address the question of whether B-ALL follows a hierarchical leukemia stem cell model.

5. Are the experiments in Figure 5 all from a single patient sample? Are the cells from a primary or passaged sample? Do the authors get similar results from other specimens?

6. Relevant to the model in Figure 6, is there any evidence to support the idea that some ALL cells are quiescent?

7. Throughout the paper the authors make statements about ALL. It would be more precise to use the term B-ALL, since hierarchical models have also been proposed for T-ALL (Cox et al., Blood 109:674; Chiu et al., Blood 116:5268) and this paper does not present data to test the hierarchical model for T-ALL.

8. The authors should also cite Williams et al. (G&D 21:2283) as this study demonstrated that that mouse B-ALLs bearing BCR-ABL could be transferred with surprisingly few cells, and did not appear to follow a cancer stem cell model.

9. It is notable that the data in Figure 2 are remarkably similar to those in Quintana et al. (2010) in making the point that cancer cells of many phenotypes are able to regenerate phenotypically diverse progeny that recapitulate the cancer from which they derived.

10. To the extent that this study concludes that the cancer stem cell model does not apply to B-ALL, the authors should not label the final column in Table 2 "stem cell frequency". Given that they argue there are no stem cells, they should label this column "LPC frequency" to be consistent with the nomenclature used in the rest of the paper.

Referee #2 (Comments on Novelty/Model System):

State-of-the art xenotransplantation model used to address a very critical question of cancer stem cells in B-ALL.

Referee #2 (Other Remarks):

In this manuscript, Rehe et al. expand upon their previous work (le Viseur et al., Cancer Cell 14:47-58), demonstrating that, unlike many other malignancies, the leukemia propagating cell in acute lymphoblastic leukemia (ALL) is frequent and phenotypically diverse. This is an interesting and important question, and the authors have undertaken a careful and thorough examination of this question. Impressively, they calculate the leukemia propagating cell frequency in many cases of primary and secondarily transplanted ALL, and with separation of subpopulations based on CD10, CD20, or CD34. These data strongly argue that leukemia propagating cells in ALL are different from AML - being more frequent and not separable by common differentiation antigens. Overall,

© EMBO

the experiments described in this manuscript are well designed and described. There are only several minor issues:

1. The data in Figure 1B reporting the total percentage of mice engrafted pooled across all patient samples does not seem to be the best way to represent this data. It might be better to report the percentage of engrafting samples where the indicated marker does not fractionate leukemia propagating activity (for example 10/12 samples engraft in table S1, and 9 of these 10 show engraftment of both CD10+ and CD10- fractions).

2. In general, it would be best to report primary and secondary engraftment data separately (as in Table 2) rather than pooled as in Figure 1B and table S1. The authors rightly point out that in vivo passage likely selects for clones that are better able to survive in xenograft host, and the most important analysis is the primary sample.

3. The comparison to the prognostic AML gene expression signature from Eppert et al. is described as distinguishing CD34+CD38- AML stem cells from CD34- AML blasts, but this signature was derived based on engrafting versus non-engrafting subpopulations. The signature from Gentles et al. was derived based on immunophentotype and some engraftment data. The text should be edited to reflect this. For example, the abstract states "... an expression signature of CD34highCD38 low leukaemia propagating cells in acute myeloid leukemia ...". Since the analysis was carried out on the Eppert et al. signature, this statement is inaccurate.

4. Figure 4B and 4C are too small to read, particularly the legend to 4B.

5. The figure legends to Supplemental Figures 1 and 2 are reversed.

6. The 33 gene set that distinguishes CD34hi and CD34lo populations should be listed in a supplemental table.

7. What methodology was used to derive the 204 gene set that distinguishes pro-B from mature B cells?

1st Revision - authors' response 13 September 2012

Response to referee #1:

Referee #1 (Comments on Novelty/Model System):

It is true that some of the content in this paper overlaps with prior papers. However I believe this paper to be the most definitive functional analysis yet. Given that this has been a controversial area, with multiple papers in high impact journals concluding based on flawed mouse models that B-ALL follows a cancer stem cell model, it is worth publishing this technically superior and more comprehensive functional analysis.

Referee #1 (Other Remarks):

Rehe et al. characterize the frequency and surface marker phenotypes of leukemia-initiating cells in B-cell acute lymphoblastic leukemia (B-ALL). This paper builds upon previously published work from the same lab (le Viseur et al., Cancer Cell 14:47). In the current study, the authors show that leukemogenic cells are present in diverse populations of B-ALL cells based upon expression of CD20, CD10 and CD34. This is true for both primary and passaged leukemia cells. Heterogeneity among leukemogenic cells was observed in both high-risk (9:22 and 11q23 mutated) and low-risk (hyperdiploid) specimens. The authors performed limit dilution experiments and concluded that B-ALL does not follow a hierarchical stem cell model. The conclusions of this paper support an emerging body of literature indicating that leukemogenic B-ALL cells are common (Diamanti et al., Leukemia, 26:376) and have diverse immunophenotypes (le Viseur et al., Cancer Cell 14:47; Kong

et al., Leukemia, 22:1207; Morisot et al., Leukemia, 24:1859). The current study extends previous studies by performing limit dilution assays of multiple subpopulations of leukemia cells. It is hard to draw any conclusion from the gene expression profile data in Figures 3 and 4 of this manuscript because of our limited knowledge regarding the genes that drive clonogenicity in B-ALL and because negative data regarding gene signatures does not prove anything. The real strength in this manuscript is the extensive functional analysis, which to my knowledge is the most comprehensive yet performed in B-ALL. The use of an improved mouse model that is more permissive for engraftment by human B-ALL cells is likely part of the reason why multiple prior studies incorrectly concluded that leukemogenic cells were rare and phenotypingally distinct in B-ALL.

1. In some cases, the limit dilution data argued against a hierarchical stem cell model, but in other cases the data were consistent with a hierarchical model (Table 2). For example, in leukemia sample EMCR1, leukemogenic cells were present in the CD20high fraction at a frequency of 1:170 and in the CD20low fraction at a frequency of 1:1700 (10-fold difference). In leukemia sample 2510, leukemogenic cells were present in the CD20high fraction at a frequency of 1:4900 and in the CD20low fraction at a frequency of 1:780 (6-fold difference). It is possible that these differences are due to technical variance. It is also possible that some leukemias are organized hierarchically, but that the markers only enrich leukemogenic cells in a subset of patients. It would be best to obtain additional data for the samples in question to determine whether differences in leukemogenic cell frequency are real or a technical aberration.

Author's response: *Indeed, this is a crucial point that deserves special attention.*

There is significant mouse to mouse variability in the human ALL NSG mouse model and we have, therefore, not only included the estimated frequency of leukaemia propagating cells but also the respective confidence intervals. The examples quoted all show overlapping confidence intervals and, therefore, clearly the estimated frequencies are not statistically different and are a product of this variability.

In the patients analysed (including the examples quoted) - because of the overlap of the confidence intervals - there was no statistically significant evidence for a hierarchy, neither in individual patients and even less when looking at all samples together. In the context of our previous work (Cancer Cell 2008) and other published data, we suggest that this may be a wider feature in B lineage ALL. However, our data do not exclude that there are individual cases of ALL or subtypes of ALL that may be different and follow a hierarchical stem cell model. We have clarified this in the manuscript (on page 13 of the revised manuscript).

Although it may be optimal to repeat the experiments with the samples in question, this would be quite challenging as it takes up to 12 months for mice to develop leukaemia after transplantation with low numbers of human cells. In the context of our other data and the positive assessment by the reviewers, we do not think that the limited gain in repeating these specific experiments would warrant such a delay in publishing our data.

2. It is not clear what the novel conclusion can be drawn from Figure 3, or how these data contribute to this manuscript. This lab has previously published microarray data comparing CD34high cells to CD34low cells (le Viseur et al., Cancer Cell 14:47). They reached the same conclusion - that decreased CD34 expression correlates with increased expression of B-cell differentiation genes, and that CD34 expression therefore distinguishes biologically different subpopulations of leukemia cells. Whether or not CD34low cells exhibit a more differentiated gene expression profile has little bearing on the relative frequency of leukemogenic cells in each subpopulation. I would recommend removing Figure 3 from this manuscript.

Author's response: *We take the reviewer's point that similar results have been described before and agree that this figure can be removed from the main article. The figure has been retained in the*

supplemental materials as it exists as counterpoint to revised Figure 3 (former Figure 4). We believe it is important to demonstrate first that real differences in expression exist between the sub-populations before demonstrating with revised Figure 3 that these differences in expression are not due to known or previously understood "self-renewal" genes. It therefore provides the positive control to the "negative" result in revised Figure 3.

3. Related to point #2, the authors put too much emphasis on the idea that a hierarchical stem cell model assumes a differentiation of leukemogenic cells into non-leukemogenic progeny according to markers similar to the differentiation of normal hematopoietic cells. For example, the sentence "The inability to identify populations by marker combinations that correspond to the well defined stages of pro-B to pre-B maturation, raises the question about the relevance of the expression levels of individual surface markers in a differentiation context". This is an overly strict interpretation of the cancer stem cell model. The mutations that transform normal cells into cancer cells might also change marker expression, alter gene expression profiles, or modify hierarchies. Therefore, a cancer can follow the stem cell model and be hierarchically organized, even if the markers that distinguish leukemogenic from non-leukemogenic cells are not the same as the markers that distinguish undifferentiated from differentiated cells in the normal lineage.

Author's response: *We agree with the referee that the malignant transformation process might disturb differentiation patterns and hierarchies and, therefore, one cannot automatically equate the normal stages of maturation with hierarchies within a cancer.*

However, in most leukaemias and cancers in which malignant stem cells have been identified, stem cell activity is mainly enriched in populations with a phenotype that resembles normal tissue stem or progenitor cells. Therefore, it is useful to analyse whether a malignant tumour shows a pattern of maturation that is similar to its tissue of origin and whether phenotypically immature are enriched for tumour propagating activity. However, we have altered the manuscript to clarify that B-ALL may have a stem cell hierarchy that may be different from that in normal B lineage maturation (on page 7 of the revised manuscript).

4. Similarly, it is not clear what the data on TERT expression really demonstrate (Fig. 4A). TERT expression levels have never been shown to distinguish tumorigenic from non-tumorigenic cells in a cancer that follows a cancer stem cell model. The fact that TERT is expressed at similar levels in CD34high and CD34low cells does not address the question of whether B-ALL follows a hierarchical leukemia stem cell model.

Author's response: *Indeed, nobody has purified cell populations based on TERT expression to demonstrate that tumour propagating activity is limited to TERT expressing cells. However, the link of TERT and long-term cellular proliferation is well established. We have also shown that TERT is a target regulated by several leukaemic fusion genes (Leukemia 24:1751-7159, 2010). Although not formal proof, these data clearly support our model which demonstrates that the different populations all have long-term leukaemia-propagating potential.*

5. Are the experiments in Figure 5 all from a single patient sample? Are the cells from a primary or passaged sample? Do the authors get similar results from other specimens?

Author's response: *These data represent pooled data from all engrafted mice transplanted with either 100-300 or 500-3000 purified ALL blasts, including mice transplanted both with cells from primary and primograft samples. The numbers of mice in each group would be otherwise too small for analysis. The actual numbers, that the individual data points in revised Figure 4 are based on, are now provided within the figure legend.*

6. Relevant to the model in Figure 6, is there any evidence to support the idea that some ALL cells are quiescent?

Author's response: *We do not have any experimental evidence for a quiescent ALL cell with leukaemia-propagating potential, however clinical experience would support the existence of such a population. In contrast to other acute leukaemias, long-term leukaemia control in ALL is significantly improved with 2 - 3 years of low intensity maintenance chemotherapy. This suggests that rare ALL blasts can survive in specific protective niches and remain quiescent for long periods of time.*

7. Throughout the paper the authors make statements about ALL. It would be more precise to use the term B-ALL, since hierarchical models have also been proposed for T-ALL (Cox et al., Blood 109:674; Chiu et al., Blood 116:5268) and this paper does not present data to test the hierarchical model for T-ALL.

Author's response: *This is a fair point. Although we suggest that our model may be applicable to other lymphoid malignancies, our data are solely based on experiments with B lineage ALL and we have modified the manuscript accordingly (including its title).*

8. The authors should also cite Williams et al. (G&D 21:2283) as this study demonstrated that that mouse B-ALLs bearing BCR-ABL could be transferred with surprisingly few cells, and did not appear to follow a cancer stem cell model.

Author's response: *An excellent suggestion and the manuscript by Williams et al. is now referenced in the manuscript (on page 15 of the revised manuscript).*

9. It is notable that the data in Figure 2 are remarkably similar to those in Quintana et al. (2010) in making the point that cancer cells of many phenotypes are able to regenerate phenotypically diverse progeny that recapitulate the cancer from which they derived.

Author's response: *We completely agree with this statement. There may be other tumour examples that do not follow a hierarchical stem cell model. We highlight this similarity and quote the work by the Morrison lab both in the Introduction (on page 4 of the revised manuscript) and the Discussion (on page 15 of the revised manuscript).*

10. To the extent that this study concludes that the cancer stem cell model does not apply to B-ALL, the authors should not label the final column in Table 2 "stem cell frequency". Given that they argue there are no stem cells, they should label this column "LPC frequency" to be consistent with the nomenclature used in the rest of the paper.

Author's response: *Point well taken. We have changed the labelling of the respective column in this table.*

Response to referee #2:

Referee #2 (Comments on Novelty/Model System):

State-of-the art xenotransplantation model used to address a very critical question of cancer stem cells in B-ALL.

Referee #2 (Other Remarks):

In this manuscript, Rehe et al. expand upon their previous work (le Viseur et al., Cancer Cell 14:47-58), demonstrating that, unlike many other malignancies, the leukemia propagating cell in acute lymphoblastic leukemia (ALL) is frequent and phenotypically diverse. This is an interesting and important question, and the authors have undertaken a careful and thorough examination of this question. Impressively, they calculate the leukemia propagating cell frequency in many cases of

primary and secondarily transplanted ALL, and with separation of subpopulations based on CD10, CD20, or CD34. These data strongly argue that leukemia propagating cells in ALL are different from AML - being more frequent and not separable by common differentiation antigens. Overall, the experiments described in this manuscript are well designed and described. There are only several minor issues:

1. The data in Figure 1B reporting the total percentage of mice engrafted pooled across all patient samples does not seem to be the best way to represent this data. It might be better to report the percentage of engrafting samples where the indicated marker does not fractionate leukemia propagating activity (for example 10/12 samples engraft in table S1, and 9 of these 10 show engraftment of both CD10+ and CD10- fractions).

Author's response: *We agree with the reviewer and have now included the percentage of engrafted samples (both for primary and primograft samples separate) as Figure 1B. The presentation of the data in the main text of the results section and the Supporting Tables S1 and S2 have been changed to reflect the suggestion raised in this and the next point by reviewer 2.*

2. In general, it would be best to report primary and secondary engraftment data separately (as in Table 2) rather than pooled as in Figure 1B and table S1. The authors rightly point out that in vivo passage likely selects for clones that are better able to survive in xenograft host, and the most important analysis is the primary sample.

Author's response: *We have now separated the data for primary and primografted samples in Figure 1B and in the supporting Tables S1 and S2. We have also adapted the results chapter accordingly and present the data as engrafted primary and primograft samples separately rather than as percentage of overall engrafted mice (on page 6 of the revised manuscript).*

3. The comparison to the prognostic AML gene expression signature from Eppert et al. is described as distinguishing CD34+CD38- AML stem cells from CD34- AML blasts, but this signature was derived based on engrafting versus non-engrafting subpopulations. The signature from Gentles et al. was derived based on immunophentotype and some engraftment data. The text should be edited to reflect this. For example, the abstract states "... an expression signature of CD34highCD38low leukaemia propagating cells in acute myeloid leukemia ...". Since the analysis was carried out on the Eppert et al. signature, this statement is inaccurate.

Author's response: *Indeed we have misinterpreted the manuscript by Eppert et al. As the referee points out, Eppert et al. describe a signature that was derived from functionally defined populations with the ability to re-establish human AML in immature mice. Most had a CD34highCD38low phenotype, however, in some samples leukaemia-propagating activity was also found in more mature progenitors. This signature was applied to expression profiles derived from phenotypically defined AML subpopulations. As most leukaemic stem cell activity in AML is detected in very immature blasts the Eppert LSC signature separated the different AML populations as described in Gentles et al. We have corrected the text accordingly (in the Results section on pages 9-11 and the Discussion section on pages 17-18 of the revised manuscript).*

4. Figure 4B and 4C are too small to read, particularly the legend to 4B.

Author's response: *We have improved the resolution of Figure 3 (former Figure 4). The merged document did not provide the clarity of the original figure. If this the editor thinks the figure is still too small we are happy to provide the figure in 172 mm width instead of 82mm.*

5. The figure legends to Supplemental Figures 1 and 2 are reversed.

Author's response: *The labelling of the Supporting Figures has been corrected and they are now labelled in order of appearance in the manuscript.*

6. The 33 gene set that distinguishes CD34hi and CD34lo populations should be listed in a supplemental table.

Author's response: The 33 gene set is n*ow included as Supporting Table S3* .

7. What methodology was used to derive the 204 gene set that distinguishes pro-B from mature B cells?

Author's response: *A description is given in Materials and Methods on page 22 "204 genes at least 3-fold mean differentially expressed between pro-B cells and mature B cell expression profiles as found in GSE19599". We have now included a reference to this in the main text for clarity (on page 8 of the revised manuscript.*

Response to referee #3:

Reviewer #3:

Comments on Novelty/Model System:

The data are not incredibly novel, however the experiments are well done, and in my opinion this type of work needs to be published in order to further shed light on which cancers are hierarchically organized and which are not.

Other Remarks:

Rehe et al study the question as to whether acute lymphoblastic leukemias are hierarchically organized similar to what has been shown for AML. They use the latest technologies and models to assess this questions and find that markers of lymphoid differentiation do not identify a population of cells with enriched engraftment potential and that some ALL samples have a high leukemia initiating activity in diagnostic unsorted samples. These studies are in line with previous studies from this group and others but are more extensive and convincing. For this reason this study will be important for the field.

One issue that should be addressed is the manner in which they use their data to state that they have shown that the leukemia is not arranged as a hierarchy. This, as with all cancer stem cell studies, cannot be stated definitively for ALLs that have a low percentage of engrafting cells in the diagnostic samples, of which they have some. What can be stated definitively is that these markers of lymphoid differentiation do not identify an enriched population suggesting that ALLs are not hierarchically organized and if they are the hierarchy does not appear to recapitulate normal differentiation. This may sound like semantics but this field is so full of overstatements and confusion that it is important to be clear about what an experiment can definitively demonstrate.

Author's response: *This is an important and highly valued suggestion! Indeed the stem cell field is riddled with "overstatements". We have, therefore, thoroughly revised all sections of the manuscript in line with the suggestions made by the reviewer to clearly avoid any "overstatements".*

A final point is that the authors seem to suggest that just because an AML "stem cell" signature is not found in ALL initiating cells that this somehow shows that this leukemia is not following a leukemia stem cell model. This statement cannot be made. Those data just suggest that AML and ALL do not engage the same programs for extensive proliferation/self-renewal. There is no a priori reason to believe this has to be the case in order for ALL to follow a stem cell/hierarchical model. This should be clarified.

Author's response: *This point follows on from the previous issue raised by reviewer 3. We have rephrased the manuscript by stating that our analysis shows that the two different CD34low and CD34high subpopulations of acute B lymphoblastic leukaemia are indistinguishable with respect to published self-renewal signatures derived from candidate AML and normal haematopoietic stem cell populations. This is in line with our observation that both populations can propagate the leukaemia. This is consistent with, but indeed not proof of, there being no stem hierarchy in ALL.*

I hope that our thorough revisions and responses are sufficient to allow acceptance of our manuscript for publication. The comments clearly have helped to make this a more readable and balanced manuscript.

2nd Editorial Decision 04 October 2012

Thank you for the submission of your revised manuscript "Acute B lymphoblastic leukaemia propagating cells are present at high frequency in diverse lymphoblast populations" to EMBO Molecular Medicine. We have now received the enclosed reports from the referee that was asked to re-assess it and as you will see, this reviewer is now supportive and I am pleased to inform you that we will be able to accept your manuscript pending the following final amendment:

- As highlighted by the Reviewer, please edit the manuscript text to improve clarity

I look forward to reading a new revised version of your manuscript as soon as possible.

Yours sincerely,

Editor
EMBO Molecular Medicine

***** Reviewer's comments *****

Referee #1:

It is a little disappointing that the authors did not add new data and that they disregarded a number of the suggestions. The text in the section entitled "Regulatory networks and candidate stem cell genes in B-ALL" remains confusing, and the authors rationale regarding these points is not clear thinking or rigorous. As indicated during the prior round of review, the observation that ALL-initiating cells do not appear to be hiearchically organized is important. However, little can be concluded based on gene expression profiles or Tert expression. In contrast to the authors' statement there is not a link between Tert and long-term cellular proliferation as mice can live for 3 generations without Tert and some cancers don't require it at all.

2nd Revision - authors' response 15 October 2012

Referee #1 (Remarks):

It is a little disappointing that the authors did not add new data and that they disregarded a number of the suggestions. The text in the section entitled "Regulatory networks and candidate stem cell genes in B-ALL" remains confusing, and the authors rationale regarding these points is not clear thinking or rigorous. As indicated during the prior round of review, the observation that ALL-

initiating cells do not appear to be hierarchically organized is important. However, little can be concluded based on gene expression profiles or Tert expression. In contrast to the authors' statement there is not a link between Tert and long-term cellular proliferation as mice can live for 3 generations without Tert and some cancers don't require it at all.

Author's response: *We greatly appreciate that referee #1 considers our functional studies, showing the lack of a hierarchy in ALL, to be important. As previously discussed, it would have indeed been nice to repeat some of the experiments; however, this would have taken up to 12 months for mice to develop leukaemia after transplantation with low numbers of human cells and would have caused an unduly delay in publishing these data.*

Regarding TERT: we completely agree with the reviewer that mice survive without Tert for three generations and that there are recombination-based mechanisms to restore telomeres in the absence of functional telomerase. However, lymphoid lineages in particular are known to telomerase expression as a prerequisite for clonal expansion. Furthermore, patients with TERT-compromising mutations suffer from severe forms of dyskeratosis congenita (dc), and recent work by the group of Steven Artandi has shown a strong link between telomere shortening and loss of self-renewal in iPS cells derived from dc patients, including those with heterozygous mutations in TERT. Moreover, TERT has additional functions not directly related to telomerase activity affecting viability and propagation of normal and malignant cells. Nevertheless, we would not necessarily suggest TERT as a strong therapeutic target in leukaemia, as telomerase deficiency can indeed be circumvented by cancer cells. This is exemplified by the observation that dc patients can develop MDS progressing towards AML. This is likely to be related to an increase in genomic instability, which may also account for the observation that loss of telomerase activity is a poor prognostic factor in CLL.

In summary, we appreciate that TERT expression in more mature lymphoid populations is, in itself, not a proof of concept for the lack of a stem cell hierarchy in ALL – indeed, we have not claimed that - but it is clearly consistent with our model. A maturation/differentiation-dependent down-regulation of TERT would have challenged our model while a complete lack of TERT would have been compatible both with the hierarchical and the stochastic model for the reasons highlighted by the reviewer.

Finally, we have re-written the Results section "Regulatory networks and candidate stem cell genes in B-ALL" and the corresponding paragraphs in the Discussion; hopefully our message is now presented more clearly.

© EMBO
