# Supplementary material for: Acute B lymphoblastic leukaemia-propagating cells are present at high frequency in diverse lymphoblast populations
Source: EMBO Mol Med. 2012 Dec 11;5(1):38–51. doi: 10.1002/emmm.201201703 (PMC3569652; doi:10.1002/emmm.201201703)
Supplement: Supplementary file 2 [file emmm0005-0038-SD2.pdf]

Table of Contents

Page 2 **Supporting Table S1:** Leukaemic engraftment of mice transplanted with purified CD10low and CD10high blasts

Page 3-4 **Supporting Table S2**: Leukaemic engraftment of mice transplanted with purified CD20low and CD20high blasts

Page 5 **Supporting Table S3:** 33 gene signature comprising of the most consistently differentially expressed genes between matched CD34high and CD34low leukaemic populations

Page 6 **Supporting Table S4:** Selected top significantly enriched genesets reflecting B cell maturation

Page 7 **Supporting Table S5:** 204 genes selected to form custom GSEA geneset based on selecting genes 3-fold upregulated in pro-B cells compared to mature B cells.

Page 8 **Supporting Figure S1:** Genes differentially expressed between CD34high and CD34low ALL blasts reflect B cell maturation.

Page 9 **Supporting Figure S2:** PCA plot generated using an AML stem cell signature (Gentles et al, 2010) that separates Lin-CD34highCD38low blasts (dark purple symbols) from more mature CD34low (pink symbols) blasts in AML but fails to detect any significant differences in self-renewal gene expression amongst the leukaemic subpopulations (black & white symbols) in B-ALL.

Page 10 **Supporting Figure S3:** PCA plot generated using a stem cell signature derived from normal haematopoietic stem cells (Eppert et al, 2011) that fails to detect any significant differences in self-renewal gene expression amongst the leukaemic subpopulations (black & white symbols) in B-ALL.

Page 11 **Supporting Figure S4:** PCA plot generated using a stem cell signature derived from normal haematopoietic stem cells (Kim et al, 2009) that fails to detect any significant differences in self-renewal gene expression amongst the leukaemic subpopulations (black & white symbols) in B-ALL.

Page 12 **Supporting Figure S5:** Serial engraftment of sample L4951

Page 13: **Supporting Figure S6:** Purity of flow sorted populations, related to Figure 3A.

Page 14 **References**

Supporting Table S1: Leukaemic engraftment of mice transplanted with purified CD10low and CD10high blasts

Patient ID	Passage #	Sorted Population	Cell dose transplanted			Total
			>50.000	10.000-50.000	<10.000	
**Primiary transplants**						
L578	primary	CD10high	2/4			
		CD10low	2/4			
L784	primary	CD10high		4/4		
		CD10low			3/4	
L831	primary	CD10high			0/4	
		CD10low			0/4	
L4945	primary	CD10high			0/4	
		CD10low			0/4	
L49101	primary	CD10high			2/4	
		CD10low			1/4	
L49120	primary	CD10high			2/4	
		CD10low			0/4	
ECMR1	primary	CD10high			3/4	
		CD10low			0/4	
ECMR2	primary	CD10high			2/4	
		CD10low			4/4	
All samples combined		CD10high				15/32 mice (47%)<br>6/8 primary samples
		CD10low				10/32 mice (31%)<br>4/8 primary samples
**Secondary transplants (primografts)**						
L4951	secondary	CD10high		4/4		
		CD10low		4/4		
ECMR1	secondary	CD10high			6/13	
		CD10low			6/13	
2510	secondary	CD10high			2/4	
		CD10low			2/4	
8849	secondary	CD10high		1/8	3/3	
		CD10low		2/8	0/3	
L803	secondary	CD10high		3/3		
		CD10low		2/3		
All samples combined		CD10high				19/35 mice (54%)<br>5/5 primary samples
		CD10low				16/35 mice (46%)<br>5/5 primary samples

Supporting Table S2: Leukaemic engraftment of mice transplanted with purified CD20low and CD20high blasts

Patient ID	Passage #	Sorted Population	Cell dose transplanted >50.000	10.000-50.000	<10.000	Total
	Primary transplants					
L754	primary	CD20high	4/4			
		CD20low	3/4			
L776	primary	CD20high			4/4	
		CD20low			4/4	
L831	primary	CD20high		0/4		
		CD20low		0/4		
L833	primary	CD20high			2/6	
		CD20low			3/7	
L835	primary	CD20high			1/3	
		CD20low			1/3	
L4945	primary	CD20high			0/4	
		CD20low			0/4	
L4951	primary	CD20high		2/3		
		CD20low		3/3		
L49101	primary	CD20high			3/4	
		CD20low			4/4	
L49120	primary	CD20high			0/4	
		CD20low			3/4	
ECMR1	primary	CD20high			4/4	
		CD20low			4/4	
ECMR2	primary	CD20high			0/4	
		CD20low			4/4	
All samples combined		CD20high				20/44 mice (45%) 7/11 primary samples
		CD20low				29/45 mice (64%) 9/11 primary samples

Patient ID	Passage #	Sorted Population	Cell dose transplanted			Total
			>50.000	10.000-50.000	<10.000	
**Secondary/tertiary transplants (primografts)**						
L727	secondary	CD20high	0/3			
		CD20low	0/4			
L736	secondary	CD20high	4/4	3/5		
		CD20low	6/6	4/5		
	tertiary	CD20high		4/4	2/2	
		CD20low		1/3	2/2	
L754	secondary	CD20high	5/6	5/9	8/10	
		CD20low	3/5	3/8	8/9	
	tertiary	CD20high		4/4	7/23	
		CD20low		4/4	9/24	
L776	secondary	CD20high			0/16	
		CD20low			0/16	
L835	secondary	CD20high			0/11	
		CD20low			2/12	
L4951	secondary	CD20high			16/16	
		CD20low			16/17	
	tertiary	CD20high			3/7	
		CD20low			3/7	
L4967	secondary	CD20high		3/3	3/3	
		CD20low		3/3	3/3	
	tertiary	CD20high			7/7	
		CD20low			8/8	
ECMR1	secondary	CD20high			6/13	
		CD20low			2/13	
2510	secondary	CD20high			2/8	
		CD20low			5/8	
All samples combined		CD20high				82/154 mice (53%)<br>6/9 primografts
		CD20low				82/157 mice (52%)<br>7/9 primografts

Supporting Table S3: 33 gene signature comprising of the most consistently differentially expressed genes between matched CD34high and CD34low leukaemic populations

Probes used in the PCA analysis 3 fold difference in expression and greater than 100 fold difference in absolute intensity in each patient pair		
**Name**	**Description**	**Average Fold Difference CD34+/CD34-**
208886_at	H1F0	3.920264452
208863_s_at	SFRS1	3.392937451
202481_at	DHRS3	3.298734434
225414_at	RNF149	3.243792811
201656_at	ITGA6	3.096603552
202242_at	TSPAN7	3.008169683
212587_s_at	PTPRC	0.331922479
234151_at	CUGBP2	0.330911086
208894_at	HLA-DRA	0.328816887
215894_at	PTGDR	0.326221706
204897_at	PTGER4	0.293586971
1556818_at	ARID1B	0.287420927
234032_at	ZCCHC7	0.283184382
203140_at	BCL6	0.28241247
219471_at	C13orf18	0.275930399
243808_at	HEL308	0.272775179
213142_x_at	LOC54103	0.257796409
1559975_at	BTG1	0.228727141
202625_at	LYN	0.197945706
202626_s_at	LYN	0.190213044
236796_at	BACH2	0.178248828
215121_x_at	IGL@ /// IGLV4-3 /// IGLV3-25 /// IGLV2-14	0.172813856
214836_x_at	IGKC /// IGKV1-5	0.096115712
209138_x_at	IGL@	0.084412027
233955_x_at	CXXC5	0.073531742
214677_x_at	IGL@ /// IGLV4-3 /// IGLV3-25 /// IGLV2-14 /// IGLJ3	0.060111035
228592_at	MS4A1	0.048957169
211430_s_at	F7 /// IFI6 /// IGH@ /// IGHG1 /// IGHG2 /// IGHG3 /// IGHM /// IGHV4-31	0.041122448
217022_s_at	IGHA1 /// IGHA2	0.028861258
221651_x_at	IGKC /// IGKV1-5 /// IGKV2-24	0.026273104
216834_at	RGS1	0.025249515
221671_x_at	IGKC /// IGKV1-5 /// IGKV2-24	0.01938833
224795_x_at	IGKC /// IGKV1-5 /// IGKV2-24	0.017208561

Supporting Table S4: Selected top significantly enriched genesets. Gene Set Enrichment analysis was run using the stand alone application from the broad institute ranked according to expression differences amongst CD34 sub-populations. The geneset library used was MSigDB C2v3 with an added custom geneset reflecting the difference between pro-B and pre-B cells genesets size was limited to between 15 and 300 genes.

NAME	SIZE	Enrichment Score	Normalised Enrichment Score	NOM p-val	FDR q-val	FWER p-val	RANK AT MAX
TARTE_PLASMA_CELL_VS_PLASMABLAST_DN	279	0.495317	4.077052	0	0	0	6652
HOFFMANN_LARGE_TO_SMALL_PRE_BII_LYMPHOCYTE_UP	84	0.544158	3.566682	0	0	0	4432
MORI_LARGE_PRE_BII_LYMPHOCYTE_UP	49	0.601528	3.466478	0	0	0	3947
3_FOLD_UP_IN_PRO_B	190	0.449552	3.453178	0	0	0	6321
MORI_IMMATURE_B_LYMPHOCYTE_DN	48	0.581616	3.351945	0	0	0	3924
SHAFFER_IRF4_TARGETS_IN_ACTIVATED_B_LYMPHOCYTE	74	0.520252	3.326989	0	0	0	6601
BASSO_B_LYMPHOCYTE_NETWORK	122	0.458727	3.308863	0	0	0	5555
TARTE_PLASMA_CELL_VS_B_LYMPHOCYTE_DN	36	-0.30328	-2.34683	0	0.008002	0.131	2397

Supporting Table S5: 204 genes selected to form custom GSEA geneset based on selecting genes 3-fold upregulated in pro-B cells compared to mature B cells

DACH1	AGPS	NCAPG	THYN1
MAPBPIP	CTBP2	SLC8A1	CCNA2
HBA1 /// HBA2	B4GALT6	E2F7	DTL
MGST1	MSRB3	KIF23	S100A8
IRX1	LOC90925	UBE2T	RNASEH2A
PXDN	BCAT1	CLEC11A	RAG2
ECT2	POLR3K	IGF2BP2	ERG
DHFR	TNFRSF1A	ADA	CENPA
MSH2	AIF1	TOM1	LOC641518
FAM64A	PGK1	CENPF	CDKN3
CLGN	ENSA	BUB1	E2F8
DEPDC1	CDCA3	ENO1	MSRB2
EFCAB2	CHRDL1	CTBP2 /// LOC642909 /// LOC645291 /// LOC645508 /// LOC649922 /// LOC650999	CEP55
AZU1	DOK4	COL5A1	DKFZP761M1511
C10orf25	KIAA1450	SPINK2	FHIT
B3GNT5	MKI67	HBD	CMTM8
CTSG	TTK	NID2	CCNB2
PIGF	IMPA2	CLC	WBP5
CDC6	NIPSNAP3B	MYB	NUSAP1
---	AURKA	SMAD3 /// MGC33556	SLC40A1
POLA1	C6orf173	STS-1	MPO
SPTA1	KIF14	LOC144481	C11orf75
TMSL8	MEST	LOC646593	HMMR
IQGAP3	PRKAR2B	RACGAP1	MPPED2
NCAPH	PTPRD	CCNB1	TPX2
GPR125	TRAPPC1	AURKB	KIF15 /// NPVF
AKAP12	DEPDC1B	ANLN	MND1
KIAA0101	LOC81691	SGOL2	LYZ
FAM112B	FLJ37673	TRIP13	GLRX
MLSTD1	CAV1	P4HA2	SAC3D1
EGFL7	CCDC34	MGST2	C10orf10
PKP4	SMAD1	CYGB	ASPM
FRMD4B	FLT3	CD109	HMHB1
GGH	NT5DC2	AKR1C3	RRM2
HBA1	GTSE1	OIP5	NPY
CDC45L	CCDC81	NEK2	KIF11
LILRA2	ZNF423	CDCA5	SOCS2
C18orf10	CPA3	KIF20A	IGFBP7
ANXA1	MST150	KIF4A	ELA2
RPUSD3	PBK	SLC22A16	RAG1
TYMS	MAGED4	MCM10	DLG7
ME3 /// PRSS23	H2AFY	EPDR1	MYLK
ZMYM6	ZNRF1	MCM4	HBB
PPBP	EXOD1	FAM72A /// LOC653820 /// LOC729533	IGLL1
FAM7A2	PRTN3	HOXA9	BIRC5
PRIM1	ARPP-21	DNTT	UHRF1
MS4A3	PRG2	MAD2L1	GINS1
TNFSF13B	CDC2	SEPP1	ZWINT
CST7	FOXM1	KIF2C	TRAF4
TSGA14	SCHIP1	CTGF	ACTN1
CDCA8	NDN	SPBC25	VPREB1

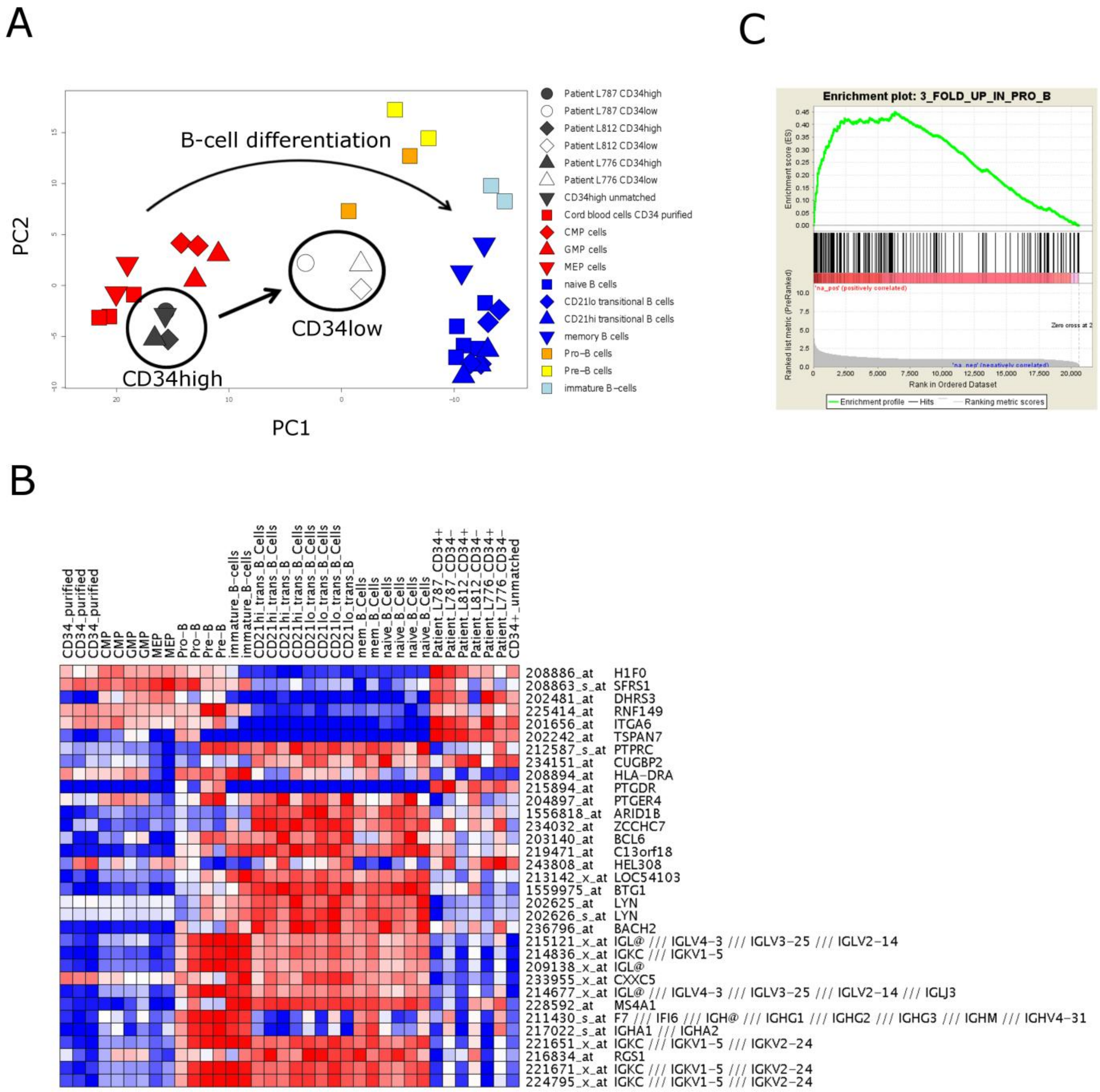

Supporting Figure S1: Genes differentially expressed between CD34high and CD34low ALL blasts reflect B cell maturation. For information on the genesets used and a list of all probes and genes see Supporting Tables S3 and S4. **A** - Principal component analysis (PCA) plot generated using a set of 33 probes which are consistently differentially expressed amongst the CD34high and CD34low populations. The progression from progenitor to mature cell is clearly distinguishable along the PC1 axis and both populations are clearly distinct. **B** - Heatmap of the expression of these 33 probes. **C** - Example gene set enrichment analysis (GSEA) showing significant enrichment in the CD34high subpopulation of a custom geneset produced to reflect the differences in expression between pro-B cells and mature-B cells; NES = 3.47 n=190 $p<0.001$ $q<0.001$.

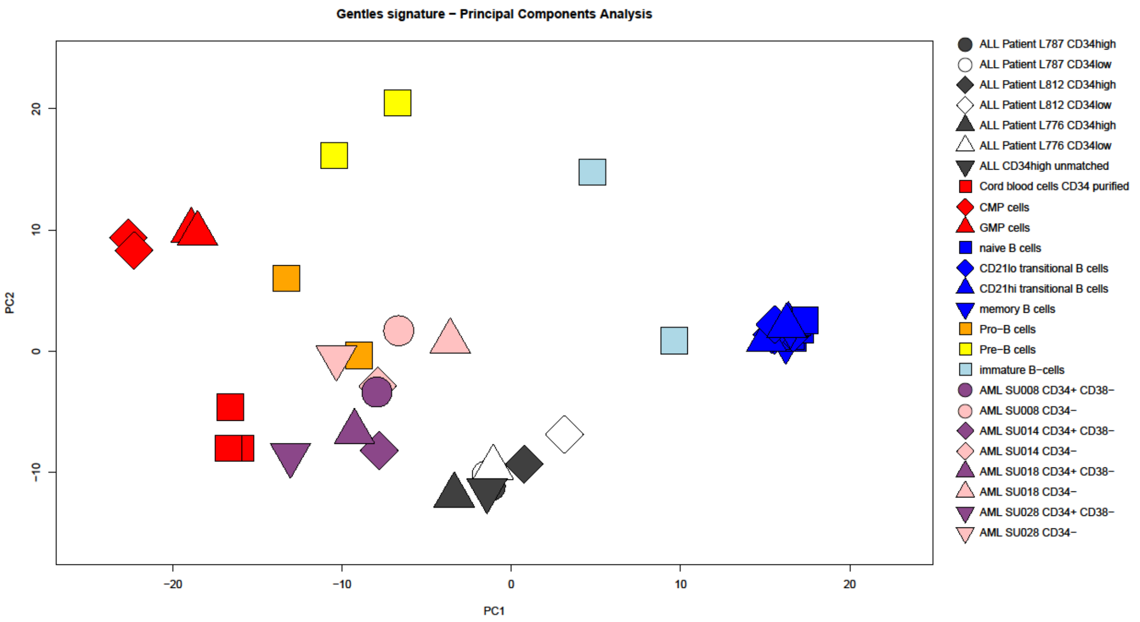

Supporting Figure S2: PCA plot generated using an AML stem cell signature (Gentles et al, 2010) that separates paired Lin-CD34highCD38low blasts (dark purple symbols) from more mature CD34low (pink symbols) blasts in AML but fails to detect any significant differences in this gene expression signature amongst the leukaemic subpopulations (black & white symbols) in B-ALL. The PCA values shown here are taken from the algorithm provided by the PCA module in TMEV4 which provides the greatest resolving power in this instance.

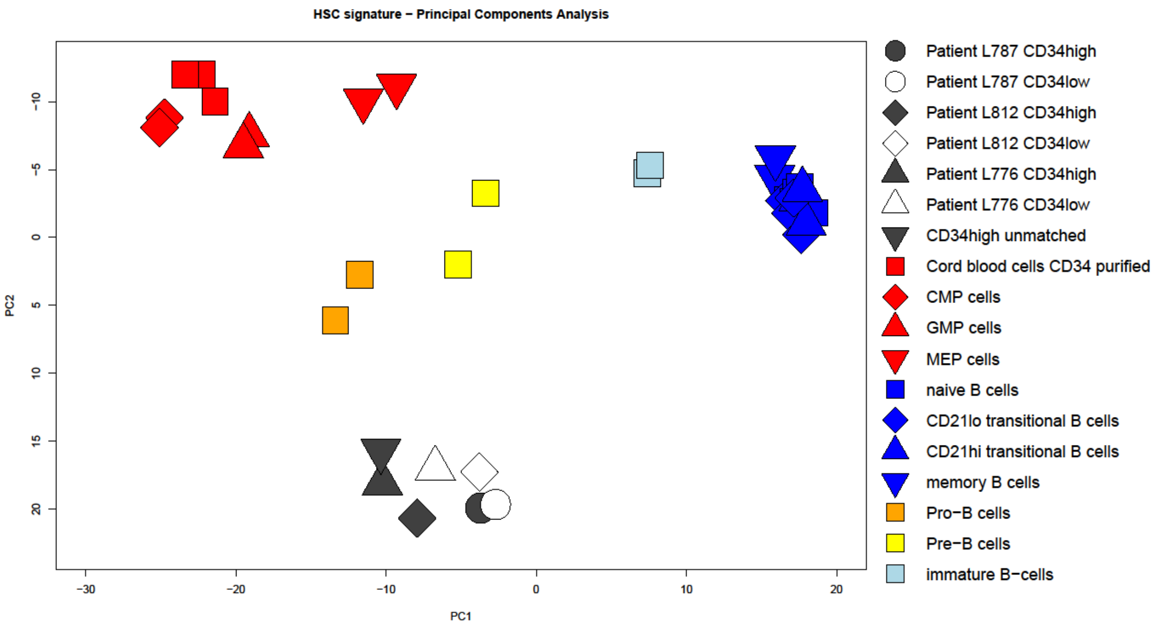

Supporting Figure S3: PCA plot generated using a stem cell signature derived from normal haematopoietic stem cells (Eppert et al, 2011) that fails to detect any significant differences in expression of this signature amongst the leukaemic subpopulations (black & white symbols) in B-ALL.

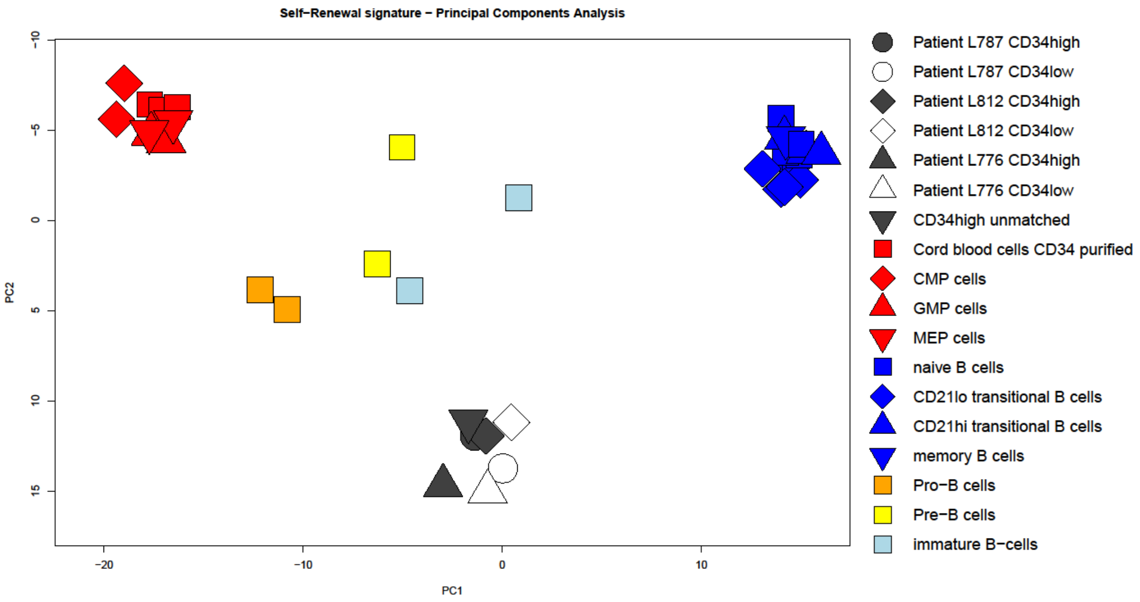

Supporting Figure S4: PCA plot generated using a stem cell signature derived from normal haematopoietic stem cells (Kim et al, 2009) that fails to detect any significant differences in self-renewal gene expression amongst the leukaemic subpopulations (black & white symbols) in B-ALL.

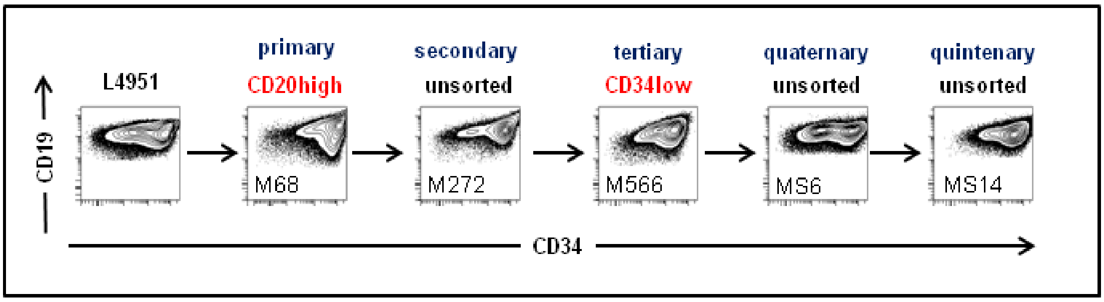

Supporting Figure S5: Serial engraftment of sample L4951 demonstrating long-term self-renewal capacity in more mature populations sorted sequentially for CD20high followed by sorting for CD34low.

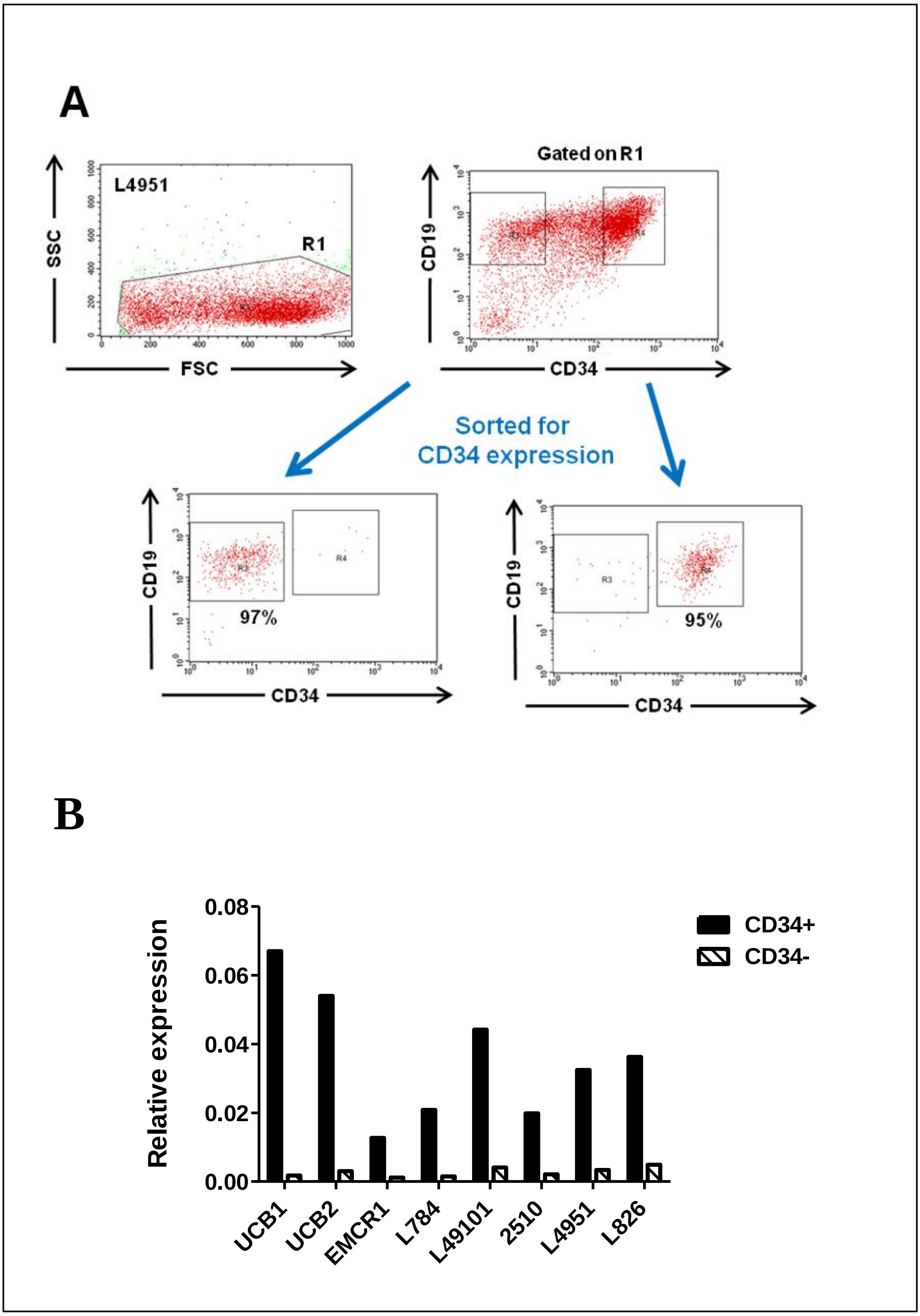

Supporting Figure S6: Purity of flow sorted populations, related to Figure 3A. **A -** Example of sorting strategy for CD34high/low leukaemic blasts. Mean purity: CD34high 88% (range 64 - 97%); CD34low 95% (range 89 -99%). **B -** Control for expression of CD34 in sorted populations showing substantially (9.6 - 38 fold) higher expression in CD34high cells.

References

Eppert K, Takenaka K, Lechman ER, Waldron L, Nilsson B, van Galen P, Metzeler KH, Poeppl A, Ling V, Beyene J et al (2011) Stem cell gene expression programs influence clinical outcome in human leukemia. Nature Medicine 17: 1086-1093

Gentles AJ, Plevritis SK, Majeti R, Alizadeh AA (2010) Association of a leukemic stem cell gene expression signature with clinical outcomes in acute myeloid leukemia. JAMA 304: 2706-2715

Kim YC, Wu Q, Chen J, Xuan Z, Jung YC, Zhang MQ, Rowley JD, Wang SM (2009) The transcriptome of human CD34+ hematopoietic stem-progenitor cells. Proceedings of the National Academy of Sciences of the United States of America 106: 8278-8283
